# Therapeutic Efficacy of Plant-Derived Exosomes for Advanced Scar Treatment: Quantitative Analysis Using Standardized Assessment Scales

**DOI:** 10.3390/ph18081103

**Published:** 2025-07-25

**Authors:** Lidia Majewska, Agnieszka Kondraciuk, Iwona Paciepnik, Agnieszka Budzyńska, Karolina Dorosz

**Affiliations:** 1 ESME Clinic, Private Practice, ul. Lwowska 1/u16, 30-548 Kraków, Poland; kursy@esmeclinic.pl; 2 Kondraciuk Clinic, Private Practice, ul. Ostrowska 6, 07-410 Ostrołęka, Poland; biuro@esmeclinic.pl; 3 Department of Family Medicine, Jagiellonian University Medical College, ul. Bocheńska 4, 31-061 Kraków, Poland; iwona.paciepnik@uj.edu.pl; 4 Małopolska Burn and Plastic Surgery Center, Os. Złotej Jesieni 1, 31-826 Kraków, Poland; 5 Biological Sciences Division, University of Chicago, Chicago, IL 60637, USA; kdorosz@uchicago.edu

**Keywords:** exosomes, plant-derived exosomes, wound healing, scar treatment, microneedling, laser therapy, skin regeneration, rose stem cell exosomes, RSCEs, case series

## Abstract

**Background**: Wound healing and scar management remain significant challenges in dermatology and aesthetic medicine. Recent advances in regenerative medicine have introduced plant-derived exosome-like nanoparticles (PDENs) as potential therapeutic agents due to their bioactive properties. This study examines the clinical application of rose stem cell exosomes (RSCEs) in combination with established treatments for managing different types of scars. **Methods**: A case series of four patients with different scar etiologies (dog bite, hot oil burn, forehead trauma, and facial laser treatment complications) was treated with RSCEs in combination with microneedling (Dermapen 4.0, 0.2–0.4 mm depth) and/or thulium laser therapy (Lutronic Ultra MD, 8–14 J), or as a standalone topical treatment. All cases underwent sequential treatments over periods ranging from two to four months, with comprehensive photographic documentation of the progression. The efficacy was assessed through clinical photography and objective evaluation using the modified Vancouver Scar Scale (mVSS) and the Patient and Observer Scar Assessment Scale (POSAS), along with assessment of scar appearance, texture, and coloration. **Results**: All cases demonstrated progressive improvement throughout the treatment course. The dog bite scar showed significant objective improvement, with a 71% reduction in modified Vancouver Scar Scale score (from 7/13 to 2/13) and a 61% improvement in Patient and Observer Scar Assessment Scale scores after four combined treatments. The forehead trauma case exhibited similar outcomes, with a 71% improvement in mVSS score and 55–57% improvement in POSAS scores. The hot oil burn case displayed the most dramatic improvement, with a 78% reduction in mVSS score and over 70% improvement in POSAS scores, resulting in near-complete resolution without visible scarring. The facial laser complication case showed a 75% reduction in mVSS score and ~70% improvement in POSAS scores using only topical exosome application without device-based treatments. Clinical improvements across all cases included reduction in elevation, improved texture, decreased erythema, and better integration with surrounding skin. No adverse effects were reported in any of the cases. **Conclusions**: This preliminary case series suggests that plant-derived exosome-like nanoparticles, specifically rose stem cell exosomes (RSCEs), may enhance scar treatment outcomes when combined with microneedling and laser therapy, or even as a standalone topical treatment. The documented objective improvements, measured by standardized scar assessment scales, along with clinical enhancements in scar appearance, texture, and coloration across different scar etiologies—dog bite, burn, traumatic injury, and iatrogenic laser damage—suggest that this approach may offer a valuable addition to the current armamentarium of scar management strategies. Notably, the successful treatment of laser-induced complications using only topical exosome application demonstrates the versatility and potential of this therapeutic modality.

## 1. Introduction

Wound healing represents a significant challenge in clinical practice, particularly when dealing with extensive injuries that can lead to scar formation. Despite advancements in wound management, achieving optimal healing with minimal scarring remains difficult [1]. Scarring not only affects a patient’s physical appearance but can also impact functionality and quality of life, highlighting the need for more effective therapeutic approaches [1].

In recent years, extracellular vesicles (EVs), including exosomes, have emerged as promising tools in regenerative medicine. Exosomes are membrane-bound nanoparticles (30–150 nm) that facilitate intercellular communication through the transfer of bioactive molecules, including proteins, lipids, and nucleic acids [2]. Initially studied primarily from mammalian sources, research has expanded to include plant-derived exosome-like nanoparticles (PDENs), which offer several advantages including cost effectiveness, accessibility, high yield, and reduced immunogenicity [3,4,5].

Plant-derived exosome-like nanoparticles have demonstrated potential in various therapeutic applications, including wound healing [6,7]. Studies have shown that PDENs from various plant sources can promote cell proliferation, enhance migration, reduce inflammation, and modulate extracellular matrix remodeling—all critical processes in effective wound healing [7,8,9,10,11,12]. These biological effects appear to be mediated through the bioactive compounds contained within PDENs, including specific proteins, lipids, and small RNAs that can influence cellular behavior [7,11,12].

The clinical translation of PDENs for wound treatment is still in its early stages, with a growing number of case studies demonstrating their potential effectiveness when combined with established techniques such as microneedling (MN) and laser therapy [13,14]. Understanding how these plant-derived nanoparticles interact with the wound healing cascade and influence scar formation is essential for optimizing their therapeutic application.

However, despite their therapeutic potential, several limitations hinder the clinical translation of plant-derived exosome-like nanoparticles (PDENs). These include significant heterogeneity in content, lack of standardized isolation and purification protocols, and unresolved challenges in ensuring quality control and batch-to-batch reproducibility. Moreover, the absence of clear regulatory frameworks specifically addressing PDEN-based therapeutics further complicates their development. Bai et al. [15] point out that isolation methods remain poorly standardized, and the reproducibility of vesicle composition and biological activity is not yet well established. In addition, regulatory frameworks tailored specifically to PDENs are lacking, and large-scale production protocols require further optimization. Addressing these limitations will be crucial to support the safe and effective clinical translation of PDEN-based therapies [15].

This paper examines the current state of knowledge regarding plant-derived exosome-like nanoparticles in wound healing and scar management. We explore the biological properties of PDENs from various plant sources, their mechanisms of action in tissue regeneration, and emerging clinical applications. Additionally, we present case studies demonstrating the potential of exosome-based therapies in treating different types of scars, including those resulting from dog bites, burns, and traumatic injuries. By integrating basic science research with clinical observations, we aim to provide insights into how plant-derived exosomes might be effectively utilized to improve wound healing outcomes and reduce scarring.

## 2. Results

### 2.1. Dog Bite Scar Treatment Outcomes

**Figure 1:**
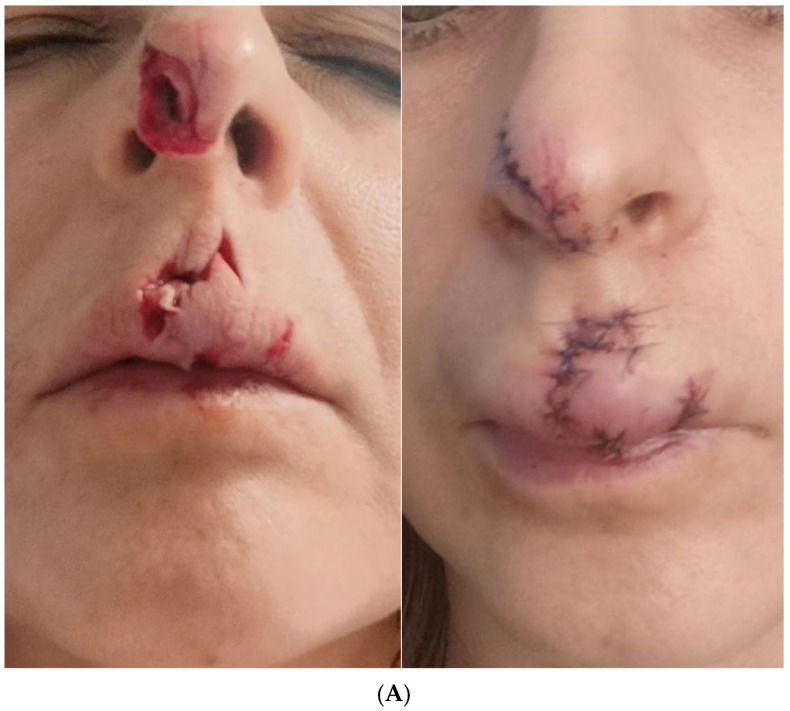

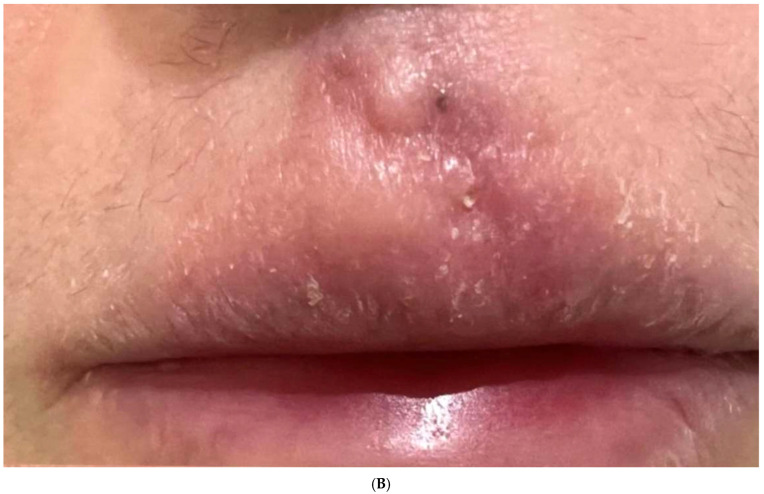

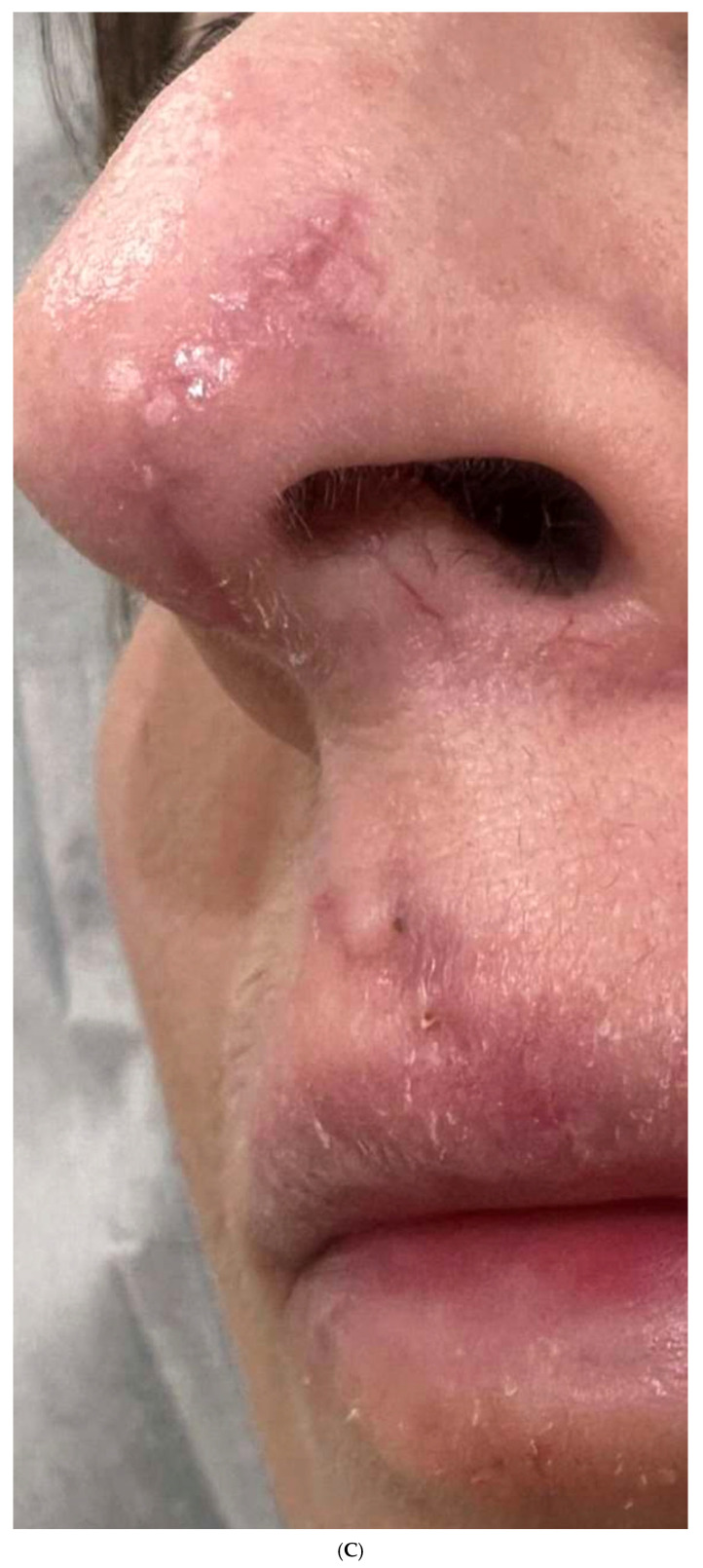

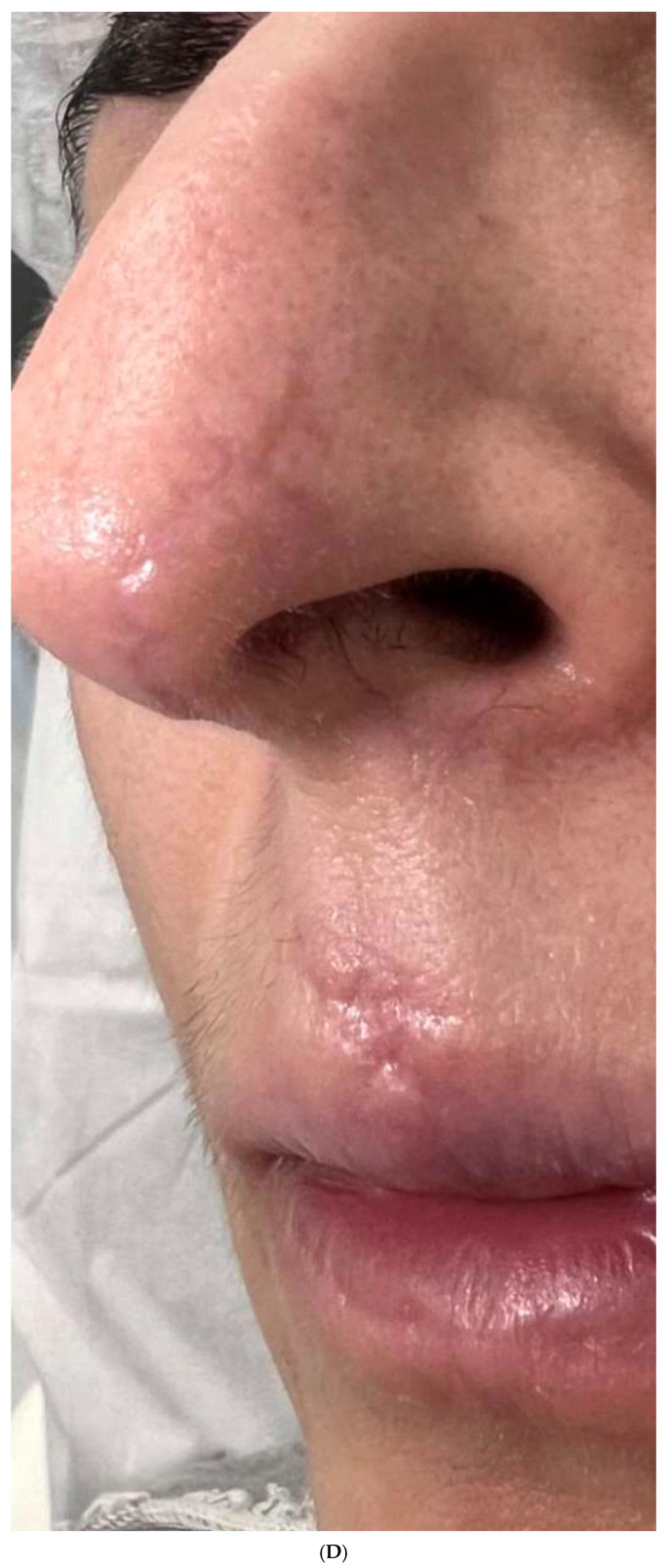

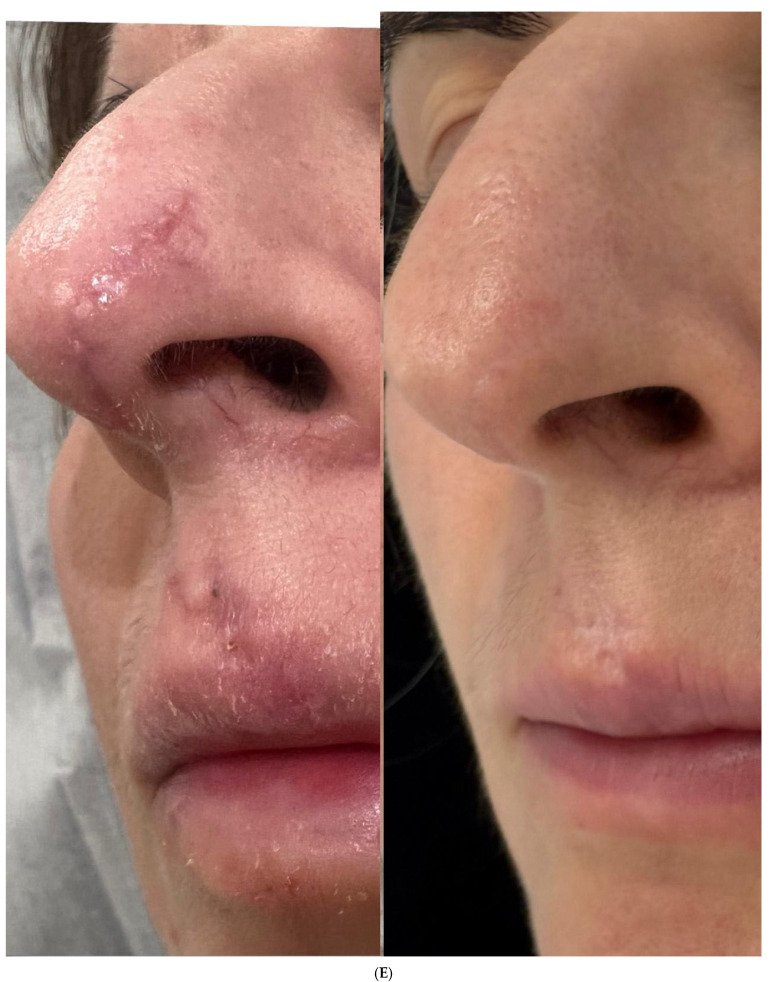
(**A**) Dog bite scar in the philtrum area before treatment (10 January 2025). (**B**) Result after first treatment session (28 February 2025). Visible reduction in scar elevation and improved texture following thulium laser and microneedling with RSCEs. (**C**) Progression after second treatment (25 March 2025). Significant reduction in erythema and further improvement in scar texture with better integration with surrounding skin. (**D**) Result after third treatment (18 April 2025). Near-complete resolution of erythema, flattening of the scar, and integration with surrounding tissue. Note the substantially improved appearance compared to baseline. (**E**) Final result a month after fourth treatment (16 May 2025). Complete resolution of erythema, flattening of the scar, and integration with surrounding tissue. Note the substantially improved appearance compared to the situation in March 2025.

The patient presented with a prominent, erythematous, and textured scar in the nose and philtrum area resulting from a dog bite. The treatment progression and outcomes were documented over the course of four sessions spanning from January to April 2025 (Figure 1A–E) with objective assessment using standardized scar scales.

The scar was located in the nose and philtrum area and exhibited noticeable erythema, elevation, and textural irregularity. The patient underwent a series of four treatment sessions from January to April 2025. Initial assessment using mVSS yielded scores for vascularity: 2 (red), pigmentation: 0 (normal), pliability: 3 (firm), and height: 2 (2–5 mm), with a total mVSS score of 7/13. The initial POSAS Observer score was 32/60, while the Patient score was 41/60, indicating significant scar-related concerns. During the first session on 31 January 2025, the patient received microneedling using a Dermapen 4.0 at needle depths of 0.2–0.4 mm, with topical application of RSCEs (0.5 mL). From day 2 post-treatment, ExoBalm was applied twice daily (morning and evening) as part of the home care protocol. Four weeks later, on 28 February 2025, the scar showed improvement, with mVSS scores for vascularity: 2 (red), pigmentation: 0 (normal), pliability: 2 (yielding), and height: 1 (<2 mm), reducing the total score to 5/13 (Table 1). The POSAS Observer and Patient scores improved to 26/60 and 35/60, respectively. The second treatment combined thulium laser (Lutronic Ultra MD, 10 J, 4 passes) with microneedling and RSCEs. By the third treatment on 25 March 2025, further significant improvement was observed, with mVSS scores for vascularity: 1 (pink), pigmentation: 0 (normal), pliability: 1 (supple), and height: 1 (<2 mm), with a total score of 3/13. The POSAS Observer and Patient scores further improved to 18/60 and 22/60, respectively. This session repeated the protocol from the second treatment. The fourth and final treatment on 18 April 2025 comprised thulium laser treatment (10 J, 4 passes) and scar acupuncture with topical application of RSCEs. Final assessment revealed significant improvement, with mVSS scores for vascularity: 1 (pink), pigmentation: 0 (normal), pliability: 1 (supple), and height: 0 (flat), and a total score of 2/13, representing a 71% improvement from baseline. The final POSAS Observer and Patient scores reached 14/60 and 16/60, respectively, representing 61% improvements in both observer assessment and patient satisfaction (Table 2). The final result showed the initially raised, irregular scar had flattened significantly, erythema had substantially decreased, scar borders had softened and integrated better with surrounding tissue, and the overall appearance was considerably less noticeable. The patient reported high satisfaction with the outcome and no adverse effects were observed throughout the treatment course.

### 2.2. Forehead Trauma Scar Treatment Outcomes

**Figure 2:**
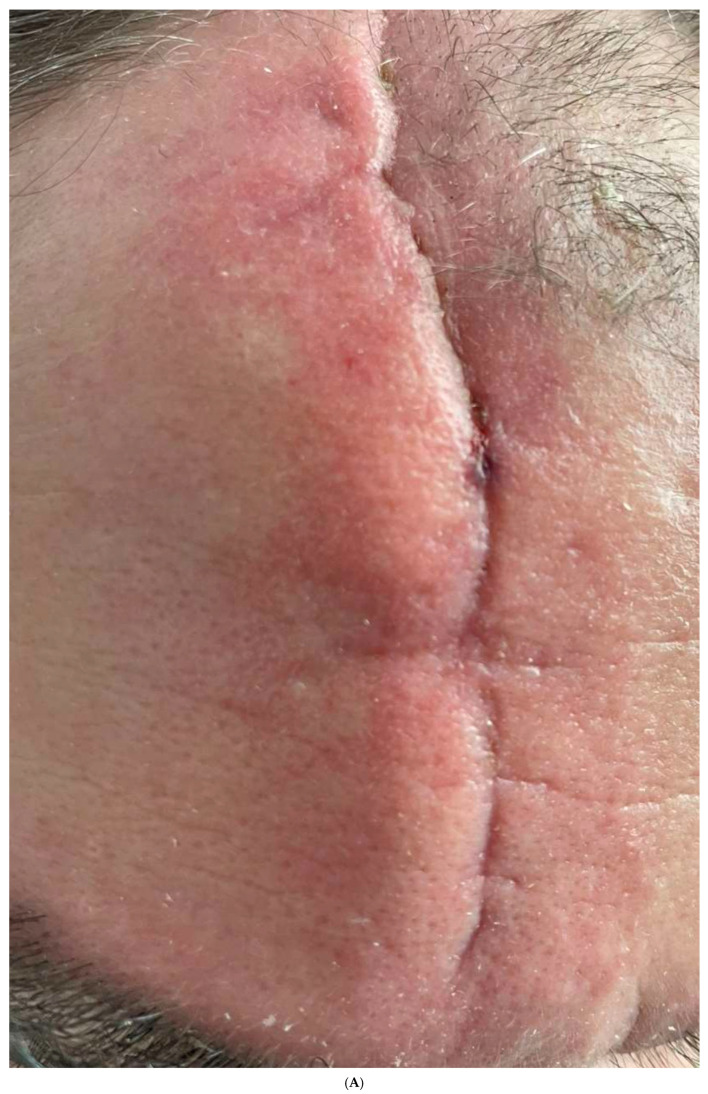

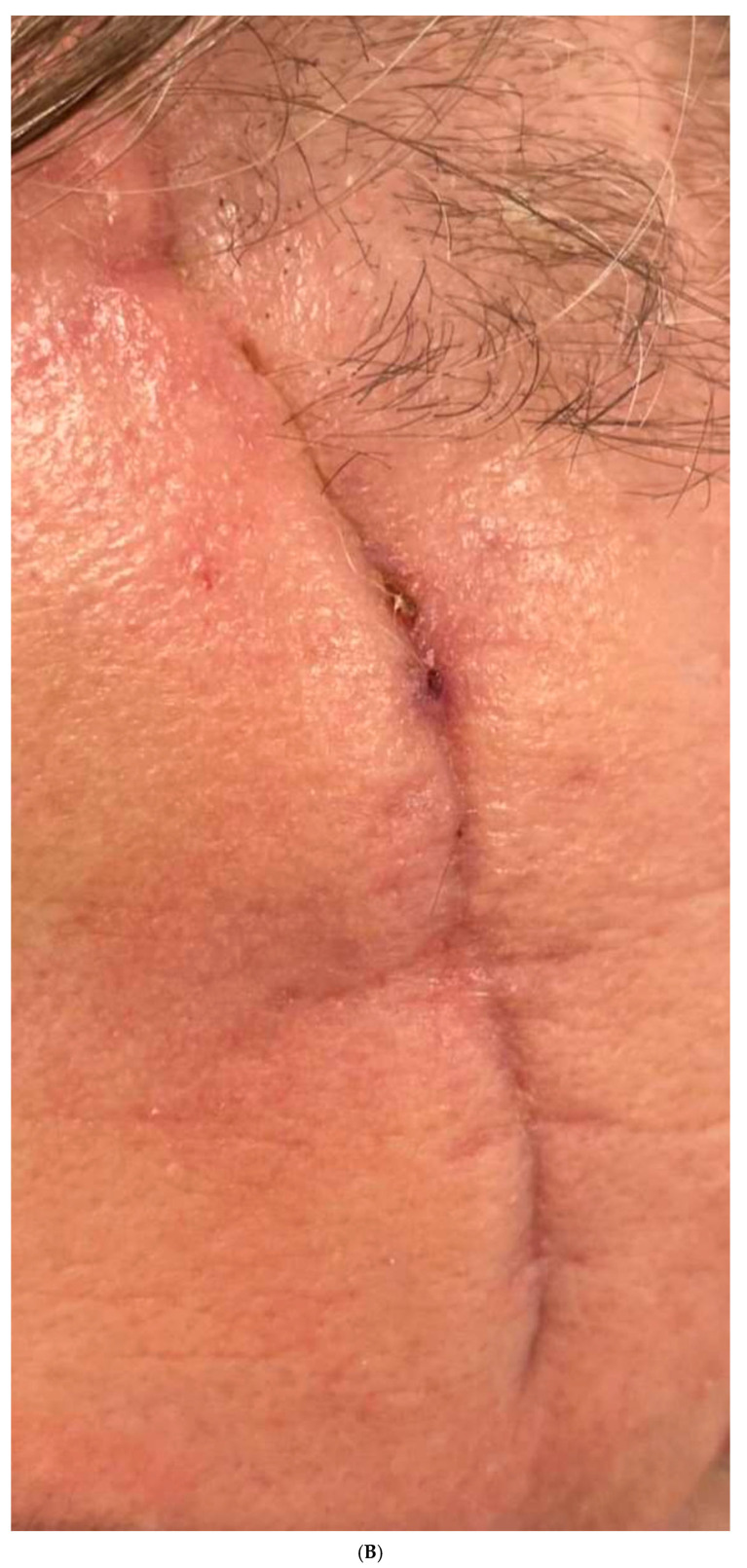

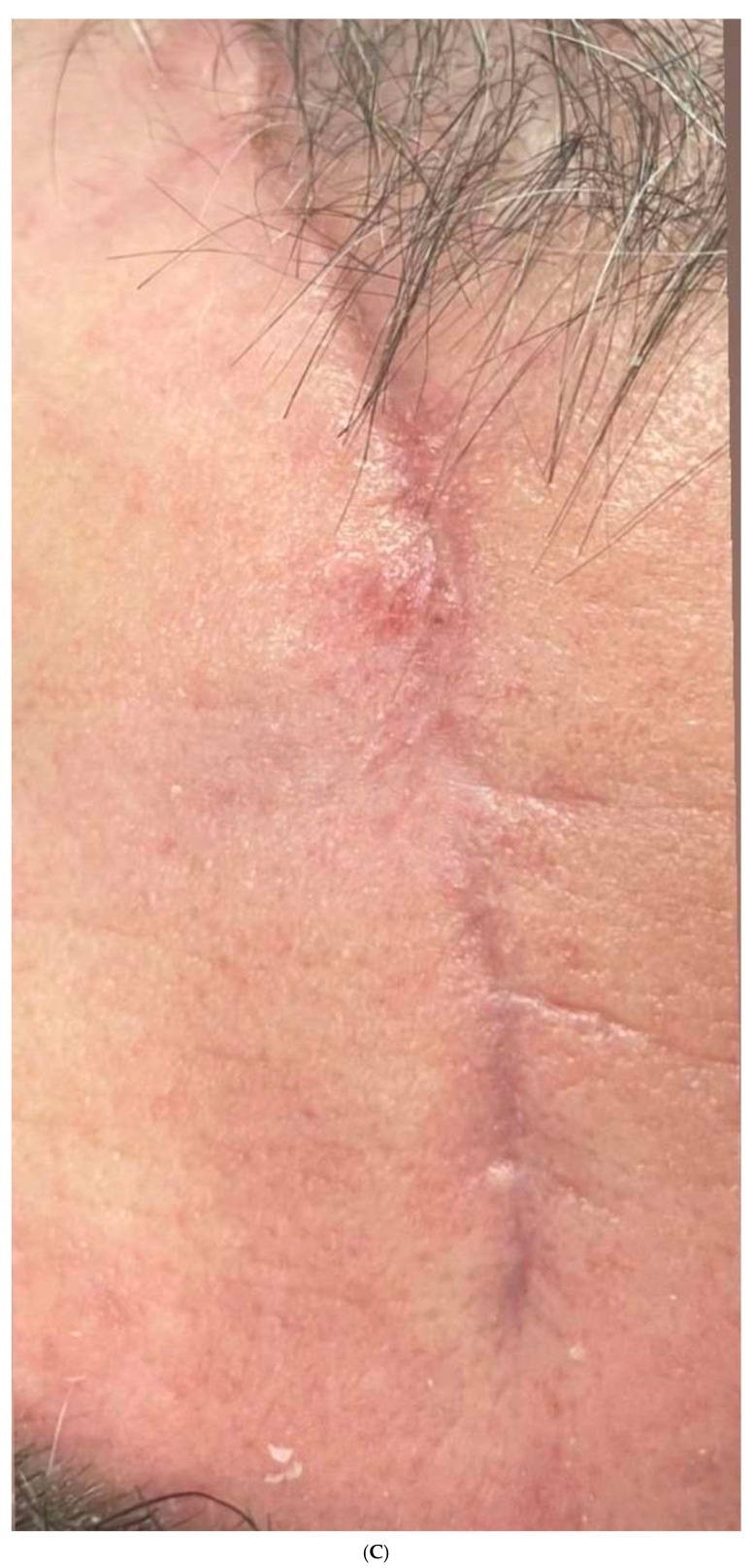

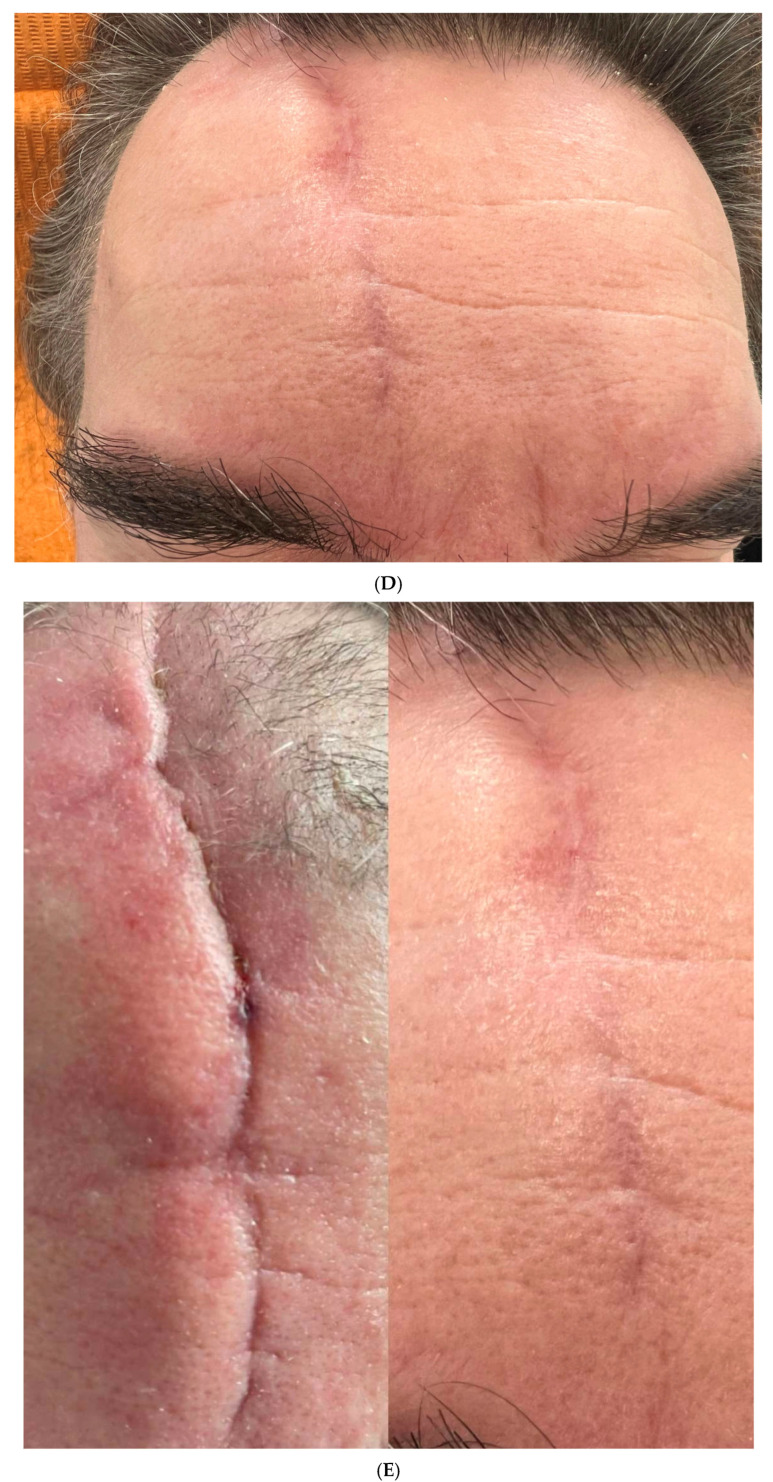
(**A**) Forehead scar resulting from car accident trauma (30 December 2024). The scar presents as elevated, erythematous, and with distinct borders. (**B**) Appearance after first treatment with thulium laser (12 J), microneedling, and RSCEs. Initial reduction in scar height and slight improvement in texture. (**C**) Progressive improvement after second treatment (28 January 2025). Note the decreased erythema and better integration with surrounding skin. (**D**) Final result after third treatment session (5 March 2025). The scar shows significant flattening, minimal erythema, and improved texture with greatly enhanced cosmetic appearance. (**E**) Side-by-side comparison of initial presentation (**left**) and final result (**right**), demonstrating the marked improvement in scar appearance after the complete treatment course.

The male patient with forehead trauma presented with a prominent, linear scar characterized by elevation, erythema, and textural irregularity. Initial assessment using mVSS yielded scores for vascularity: 2 (red), pigmentation: 0 (normal), pliability: 3 (firm), and height: 2 (2–5 mm), with a total mVSS score of 7/13. The initial POSAS Observer score was 33/60, while the Patient score was 42/60, indicating significant cosmetic and functional concerns. After the first treatment combining thulium laser, microneedling, and RSCEs, modest improvement was observed primarily in scar height and pliability. Following the second treatment, the mVSS assessment showed notable improvement, with scores for vascularity: 1 (pink), pigmentation: 0 (normal), pliability: 2 (yielding), and height: 1 (<2 mm), reducing the total score to 4/13 (Table 3). The POSAS Observer and Patient scores improved to 24/60 and 31/60, respectively. By the third and final treatment, the scar exhibited significant flattening and reduced erythema, with a final mVSS score of 2/13, representing a 71% improvement from baseline. The final POSAS Observer and Patient scores reached 15/60 and 18/60, respectively, demonstrating a 55% and 57% improvement in observer assessment and patient satisfaction, respectively (Table 4). Photographic documentation confirmed these improvements, with the scar displaying notably better integration with surrounding skin, decreased visibility, and normalized skin texture compared to the pre-treatment state (Figure 2A–E).

### 2.3. Hot Oil Burn Treatment Outcomes

**Figure 3:**
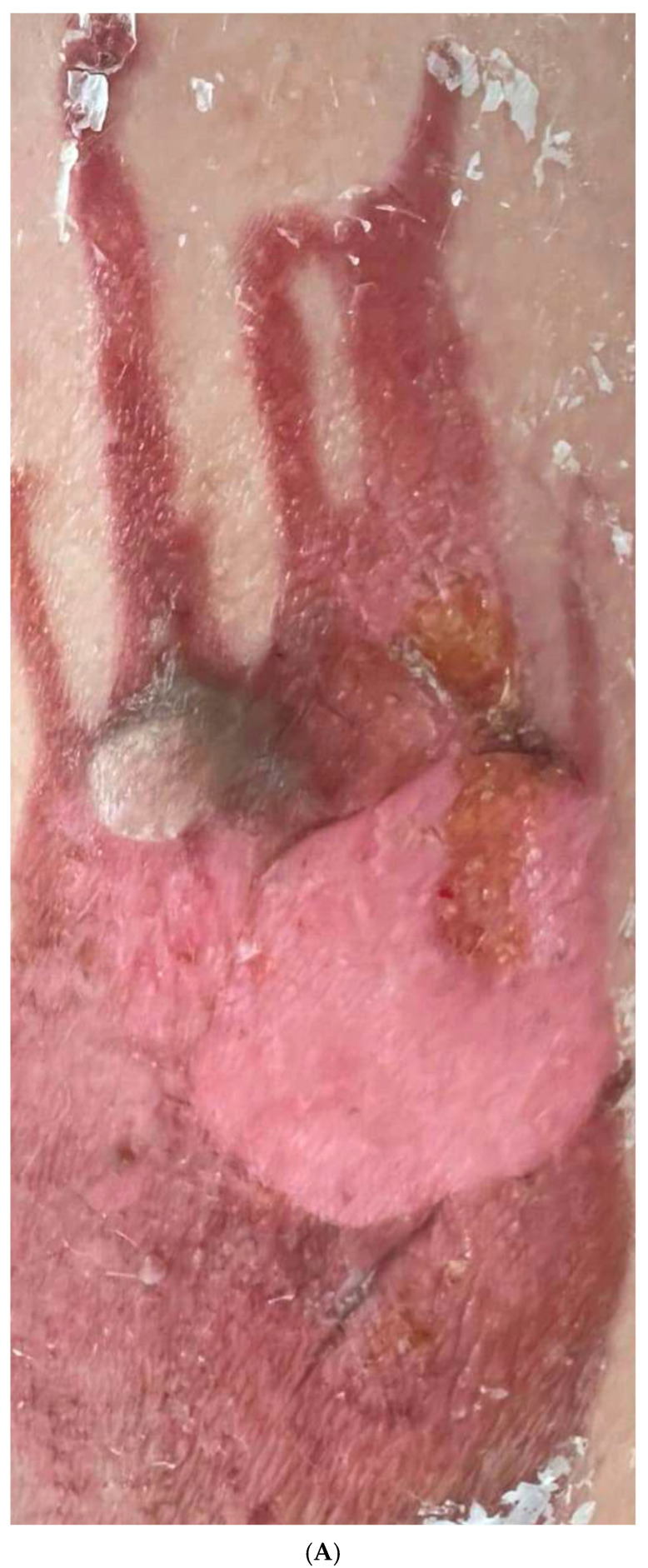

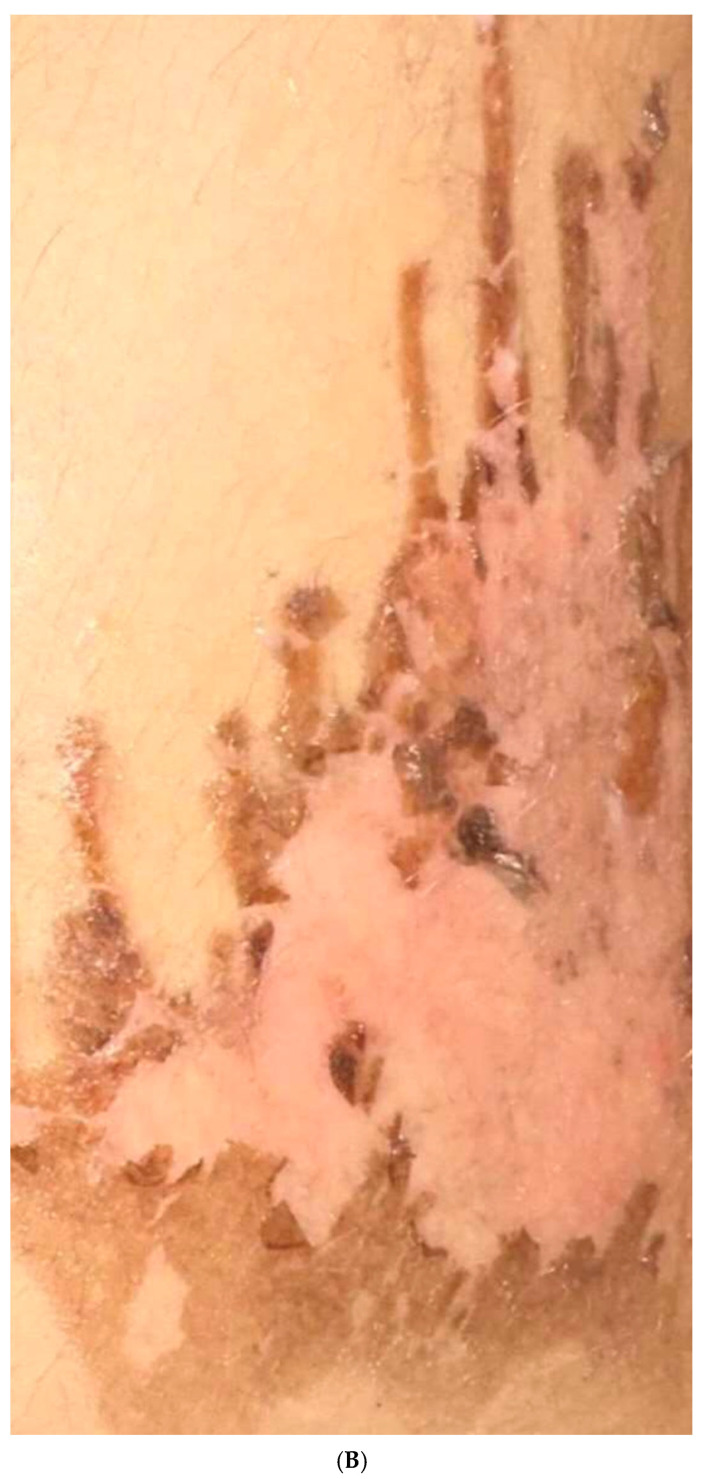

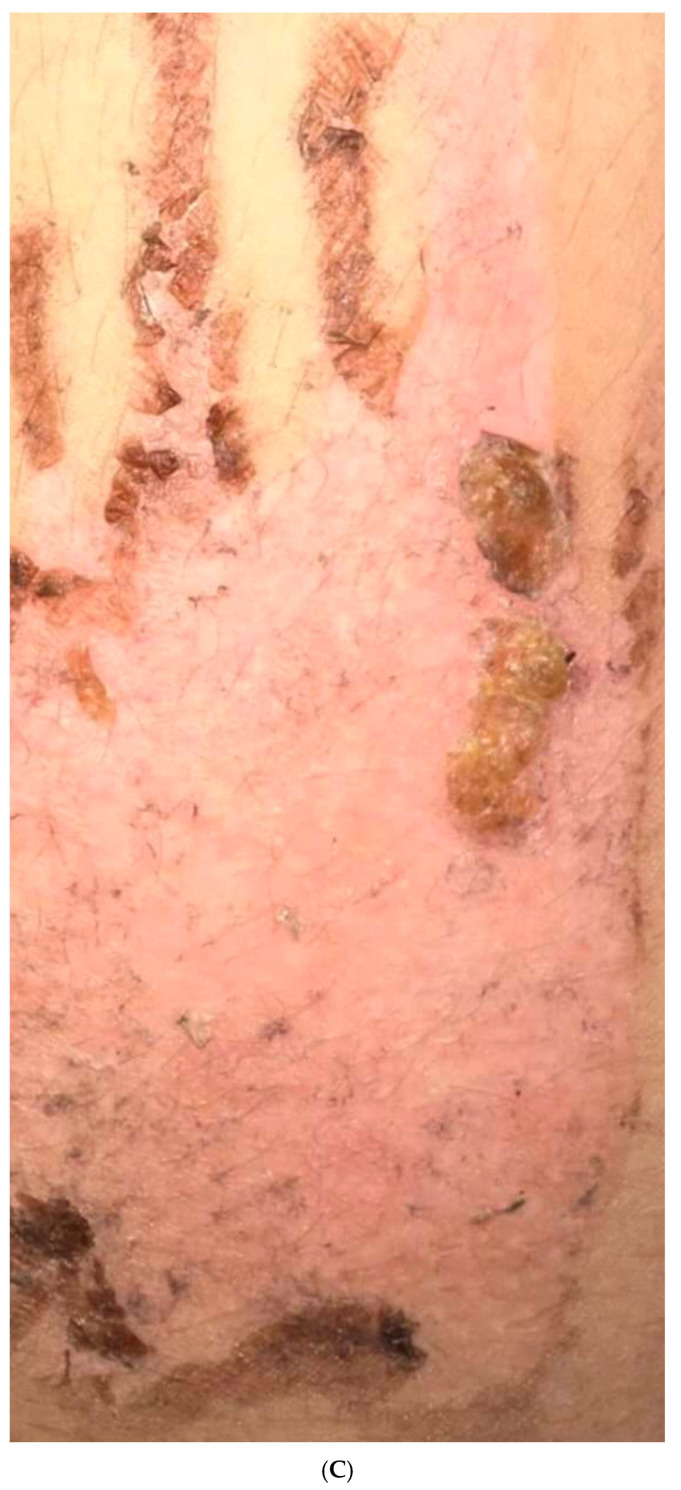

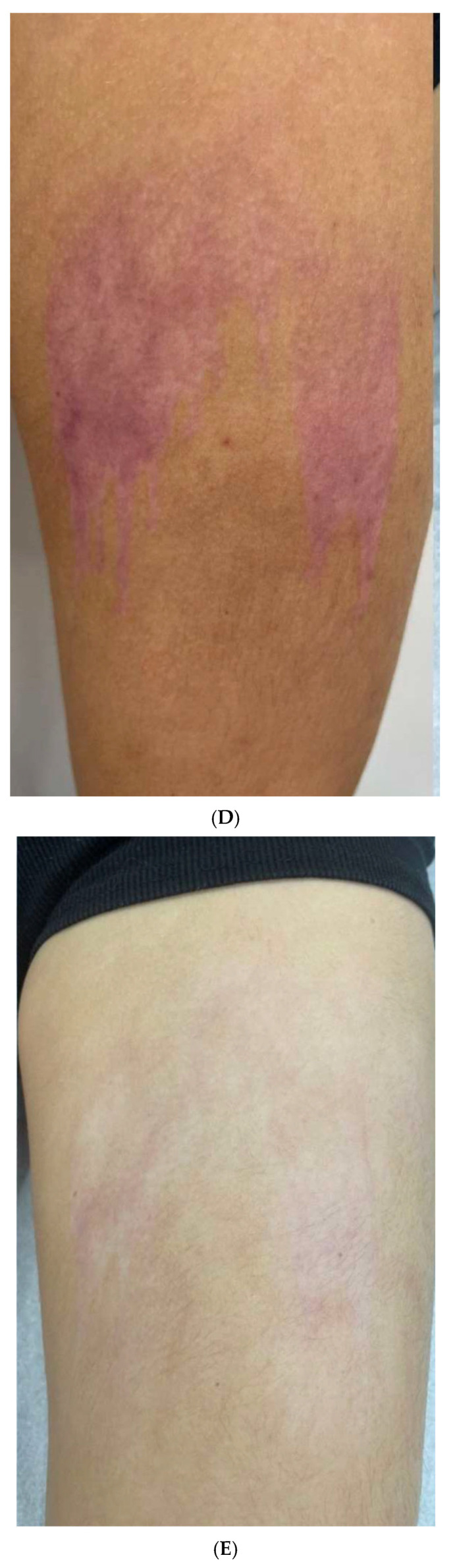

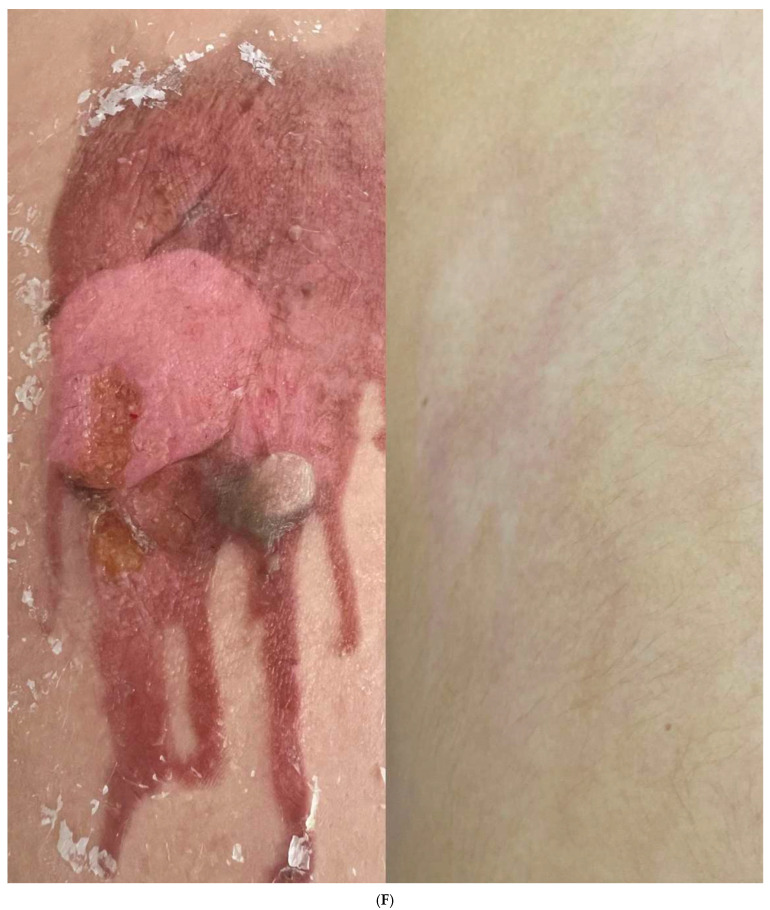
(**A**) Initial presentation of hot oil burn on the thigh (26 October 2024). Note the severe erythema, blistering, and damaged skin integrity. (**B**) Status after first treatment (31 October 2024) with thulium laser (8 J) and microneedling with RSCEs. Initial wound healing is evident with decreased erythema. (**C**) Progression after second treatment (28 November 2024). Significant improvement in tissue regeneration and continued reduction in erythema. (**D**) Appearance after third treatment (20 December 2024). Near-resolution of erythema with greatly improved skin texture. (**E**) Final result after fourth treatment (10 February 2025). Near-complete resolution with minimal residual changes and excellent color match with surrounding skin. (**F**) Comparative image showing the dramatic improvement from initial injury (**left**) to final result (**right**), demonstrating the efficacy of combined treatment with RSCEs.

The patient with a hot oil burn presented with an acute injury characterized by severe erythema, blistering, and compromised skin integrity. Initial assessment using the mVSS revealed scores for vascularity: 3 (purple), pigmentation: 1 (hypopigmentation), pliability: 3 (firm), and height: 2 (2–5 mm), with a total mVSS score of 9/13. The initial POSAS Observer score was 45/60, with a Patient score of 49/60, reflecting the severity of the injury. After the first treatment combining thulium laser therapy with microneedling and RSCEs, noticeable improvement was observed in wound healing with decreased erythema and initial tissue regeneration. Following the second treatment, the mVSS scores improved to vascularity: 2 (red), pigmentation: 1 (hypopigmentation), pliability: 2 (yielding), and height: 1 (<2 mm), reducing the total score to 6/13. The POSAS Observer and Patient scores improved to 32/60 and 37/60, respectively. The third treatment produced further significant improvement, with continued reduction in erythema and enhanced texture. By the fourth and final treatment, significant resolution was observed with near-normal skin appearance. The final mVSS assessment showed scores for vascularity: 1 (pink), pigmentation: 0 (normal), pliability: 1 (supple), and height: 0 (flat), with a total score of 2/13, representing a 78% improvement from baseline (Table 5). The final POSAS Observer and Patient scores reached 11/60 and 14/60, respectively, demonstrating a 76% and 71% improvement, respectively (Table 6). Photographic comparison between the initial injury and final result revealed near-complete resolution, with minimal residual texture changes and excellent color match with surrounding skin, suggesting that this combination therapy may be particularly effective for acute burn management (Figure 3 A–F).

### 2.4. Facial Laser Treatment Complications Case

A patient presented with significant adverse effects following a combined erbium-YAG and thulium laser treatment originally intended to improve skin quality. The patient experienced immediate complications including severe erythema, edema, textural irregularities, and persistent discomfort across the right cheek area. Within 24 h of the procedure, burn blisters developed on the treated area, and within several days, wounds appeared along the jawline, indicating significant tissue damage beyond typical post-procedural inflammation.

Initial assessment using mVSS revealed elevated scores for vascularity (3—purple), pigmentation (1—hypopigmentation in areas), pliability (3—firm), and height (1—<2 mm), with a total mVSS score of 8/13. The POSAS Observer score was 42/60, and the Patient score was 47/60, reflecting significant cosmetic and functional concerns.

Conventional management with topical antibiotics (Fusacid H), corticosteroids (Pimafucort), and hydrogel dressings in the acute phase, followed by I-PRF injection and post-inflammatory hyperpigmentation treatments (Alantan Plus, La Roche Posay Mela B3) produced limited improvement over the first six weeks. Persistent erythema, textural irregularities, and emerging post-inflammatory hyperpigmentation remained significant concerns.

At six weeks post-injury (19 March 2025), the decision was made to apply RSCEs in the form of a mask, which was continued for three consecutive days. Notably, the patient declined the use of a Dermapen or laser therapy due to concerns about further complications. The patient was provided with ExoBalm for home use, which she applied twice daily. At the follow-up appointment on 10 April 2025, the improvement was so significant that the planned Dermapen treatment was deemed unnecessary, and the recommendation was simply to continue with the ExoBalm application and strict photoprotection.

According to patient reports and clinical assessment, significant improvement was observed after initiating RSCE treatment, with accelerated healing, improved skin texture, and marked reduction in post-inflammatory hyperpigmentation, even without the use of microneedling or laser therapy as had been employed in other cases. Final assessment showed significant improvement, with the mVSS score reduced to 2/13, representing a 75% improvement from baseline (Table 7). The final POSAS Observer and Patient scores reached 13/60 and 16/60, respectively, demonstrating approximately 70% improvement in both clinical assessment and patient satisfaction (Table 8).

**Figure 4:**
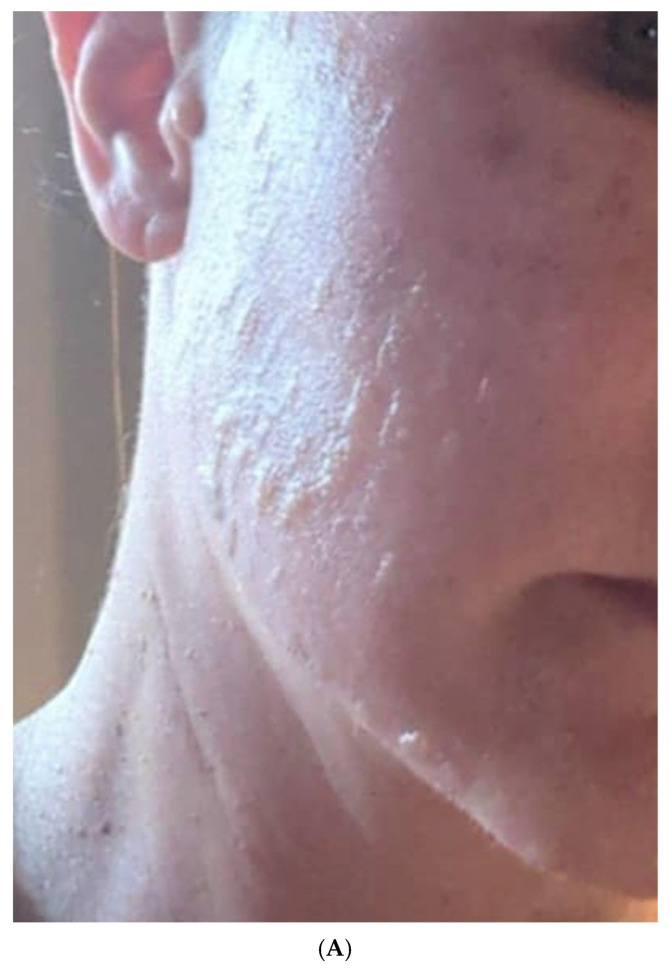

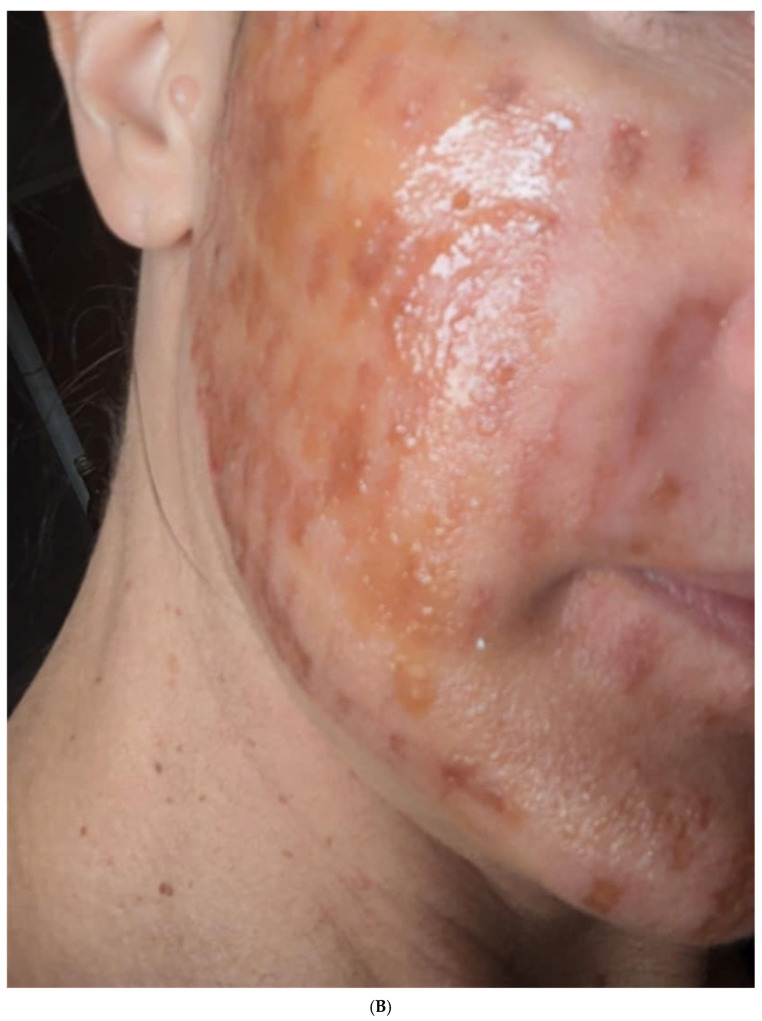

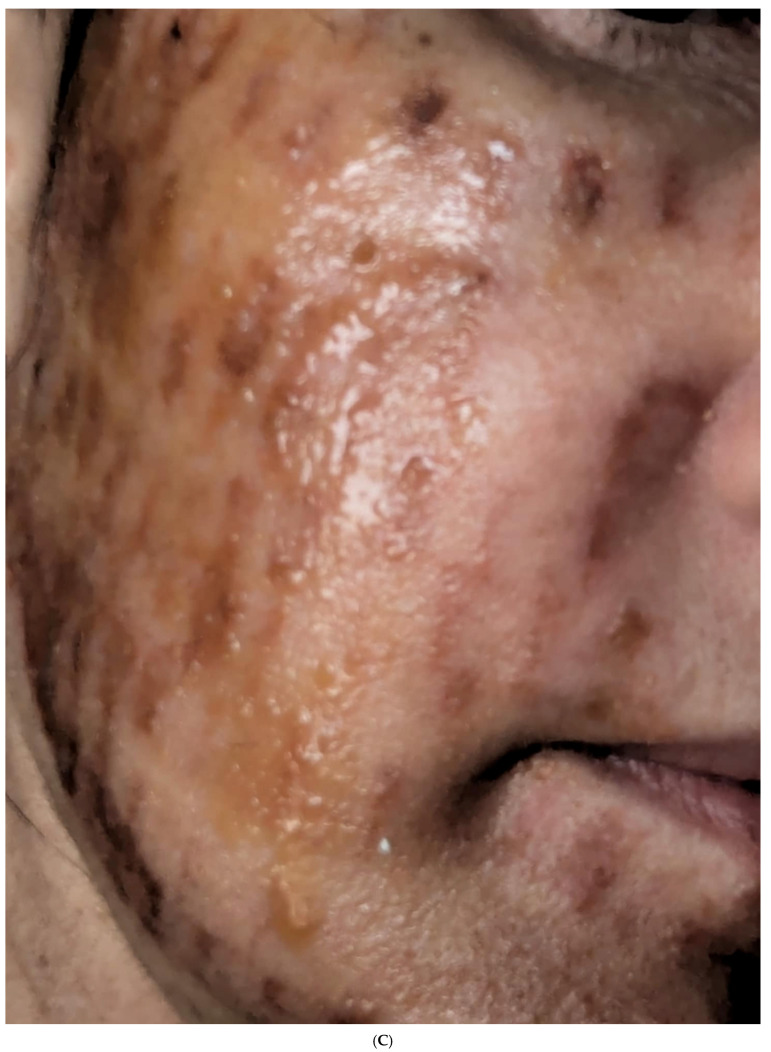

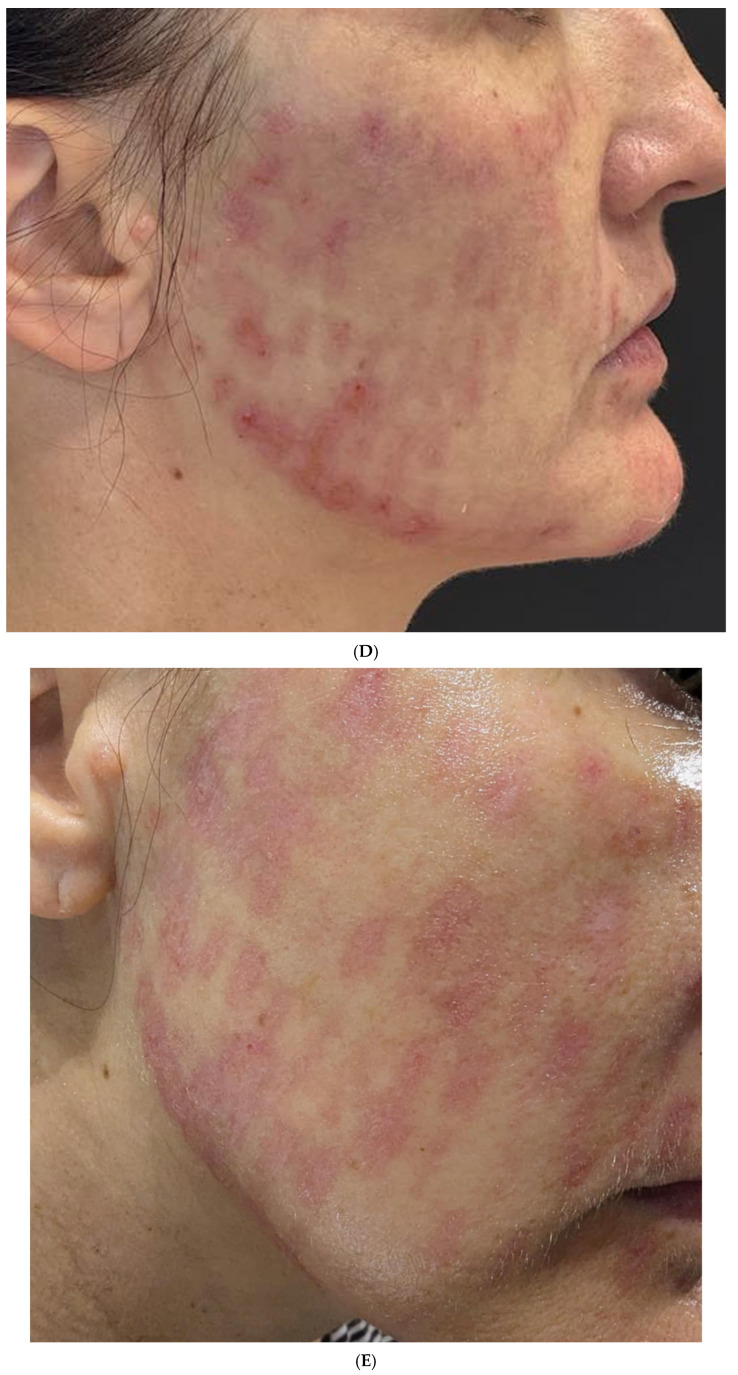

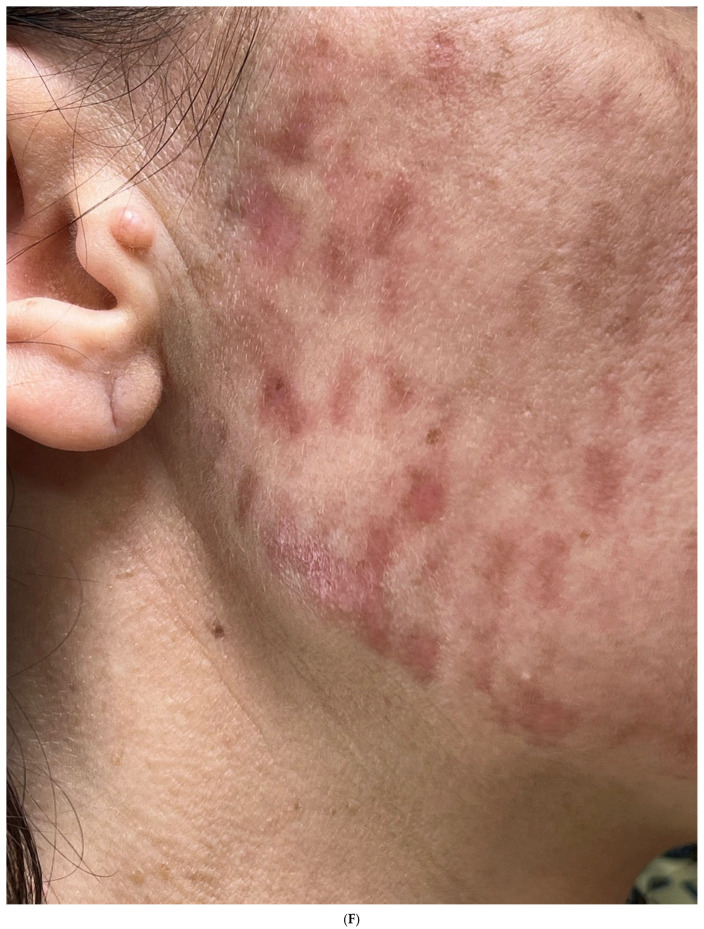

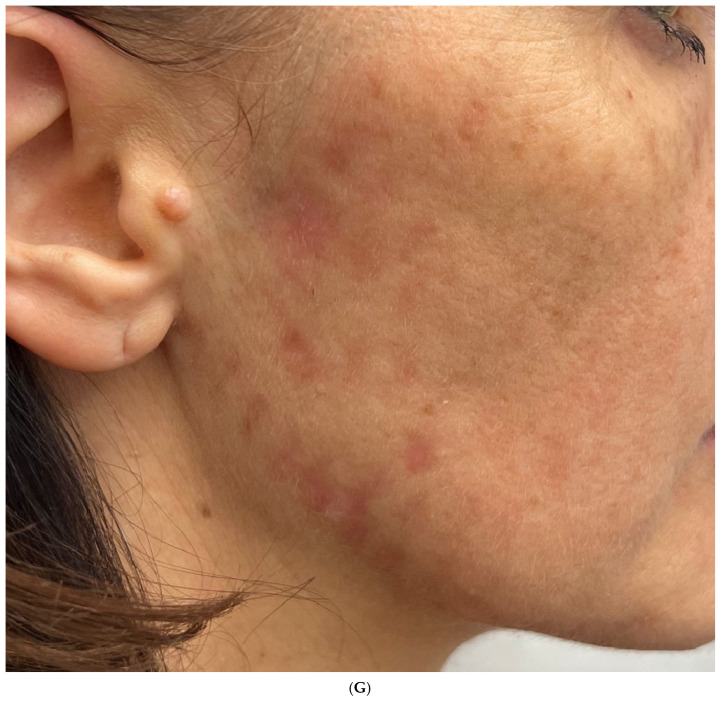
(**A**) Initial presentation 24 h after the laser procedure (1 February 2025) showing severe erythema, edema, and early blister formation following application of Fusacid H and Pimafucort by the dermatologist. (**B**) Initial management phase (1 February 2025) with hydrogel and manuka honey dressings showing significant facial inflammation and skin barrier disruption. (**C**) Early post-injury phase (4–5 days after laser treatment). Close-up view of right cheek during early treatment phase with Manuka honey dressings showing persistent erythema and early textural changes. (**D**) Eight days post-injury (8 February 2025). Note the diffuse, well-demarcated erythematous areas across the cheek before treatment with injectable PRF (one session). (**E**) Ten days post-injury and two days after I-PRF injection (10 February 2025). Close-up view of post-laser treatment complications showing detailed pattern of erythema with textural irregularities along the jawline and cheek area. (**F**) Patient photographed at six weeks post-injury (19 March 2025) at the initiation of RSCE mask treatment, showing persistent erythema and post-inflammatory hyperpigmentation before beginning the exosome protocol. (**G**) Final result (9 May 2025) after completion of RSCE treatment protocol showing significant improvement in erythema, texture, and hyperpigmentation, with near-resolution of the iatrogenic laser injury, achieved with exosome mask treatment and ExoBalm application without additional device-based interventions.

This case highlights the potential application of plant-derived exosome-like nanoparticles in managing iatrogenic complications from aesthetic procedures, particularly in addressing the challenging combination of tissue damage, textural irregularities and post-inflammatory hyperpigmentation following laser treatment adverse events. Notably, this case demonstrates that significant improvement can be achieved with RSCEs applied as a topical mask and maintenance cream, even without the adjunctive use of microneedling or laser therapy that was employed in the other cases in this series. The photographic documentation of the treatment course is presented in Figure 4A–G, illustrating the progression of healing and tissue regeneration throughout the therapeutic intervention period.

#### Summary of Objective Improvements

Across all four cases, the median mVSS improvement was 73% (IQR 71–76.5%), the median POSAS Observer improvement was 62.5% (IQR 55–72.5%), and the median POSAS Patient improvement was 63.5% (IQR 58.5–68.5%). Although the small sample size limited statistical power, Wilcoxon signed-rank tests comparing baseline to final scores showed a consistent trend toward significant improvement in both mVSS (*p* = 0.068) and POSAS scores (Observer *p* = 0.068; Patient *p* = 0.068).

### 2.5. Objective Assessment of Scar Improvement

The incorporation of standardized scar assessment scales, specifically the modified Vancouver Scar Scale (mVSS) and the Patient and Observer Scar Assessment Scale (POSAS), provided objective metrics to quantify the improvements observed in all cases. The progressive reduction in mVSS scores from baseline to after the final treatment represents a significant improvement, primarily driven by changes in vascularity, pliability, and height parameters.

Similarly, the POSAS scores showed substantial improvements in both observer and patient assessments, indicating that the clinical improvements were meaningful from both perspectives. The patient component of POSAS is particularly valuable as it captures subjective experiences like pain and itching that may not be evident in photographs or clinician assessments.

The consistent improvement across multiple standardized metrics reinforces the visual evidence provided by photographic documentation and suggests that the combination of RSCEs with microneedling and laser therapy produced clinically significant improvements in scar quality. This multi-parameter approach to assessment aligns with current recommendations for comprehensive scar evaluation in clinical research.

### 2.6. Observed Patterns Across Cases

Preliminary analysis across the documented cases revealed several consistent patterns:

1. Progressive Improvement: All cases demonstrated gradual improvement with each successive treatment, suggesting a cumulative effect of the exosome therapy when combined with microneedling and/or laser treatment.
2. Multidimensional Improvement: Enhancement was observed across multiple parameters, including scar elevation, texture, erythema, and integration with surrounding tissue.
3. Complementary Effects: The combination of physical interventions (microneedling, laser) with biological therapy (RSCEs) appeared to yield synergistic benefits for scar improvement.
4. Absence of Adverse Effects: No significant adverse reactions or complications were documented in any of the cases, suggesting a favorable safety profile for the combined treatment approach.
5. Efficacy of Standalone Topical Application: The facial laser complication case demonstrated that RSCEs can produce significant improvements even when applied only topically without device-based interventions, suggesting versatility in treatment approaches based on patient needs and clinical circumstances.

These preliminary observations provide a foundation for more systematic investigation into the efficacy of plant-derived exosome-like nanoparticles in scar management and wound healing.

## 3. Discussion

Wound healing and scar management continue to represent significant challenges in clinical practice, with current therapeutic modalities often producing suboptimal outcomes [1]. A comprehensive review of the literature revealed that the majority of published studies investigating exosome-based therapies for wound healing and scar correction predominantly focus on exosomes derived from human tissues, particularly mesenchymal stem cells (MSCs) from adipose tissue (ADSCs). Studies by Li et al. [16], Xiong et al. [17], and Li et al. [18] demonstrated that human adipose-derived stem cell exosomes can effectively promote tissue regeneration, modulate the inflammatory response, and improve scar appearance.

Despite their promising therapeutic potential, regulatory frameworks in many countries significantly restrict or entirely prohibit the clinical application of human-derived exosomes, particularly for cosmetic or aesthetic purposes. According to the European Commission’s Regulation (EC) No 1223/2009 on Cosmetic Products [19], human-derived cellular or tissue components are prohibited from use in cosmetic products. Similar restrictions exist in various international jurisdictions, limiting the practical implementation of these therapies despite their documented efficacy.

In this regulatory context, plant-derived exosome-like nanoparticles (PDENs) present a compelling alternative. Rose stem cell-derived exosomes (RSCEs) are particularly noteworthy in this regard, as they offer similar regenerative properties while circumventing many of the regulatory challenges associated with human-derived products. Won et al. [20] demonstrated that RSCEs possess biological activities relevant to skin regeneration, including stimulation of fibroblast proliferation and modulation of inflammatory responses, suggesting mechanisms potentially analogous to those observed with human MSC-derived exosomes. These preclinical data [20] suggested that RSCEs contain Let-7 family miRNAs and over 200 proteins that may enhance fibroblast proliferation, modulate inflammation (e.g., IL-6 reduction), and influence extracellular matrix remodeling—mechanisms supported by the documented improvements in our cases. These findings align with observed reductions in erythema, improved scar texture, and accelerated healing, providing a plausible biological basis for the clinical outcomes. Furthermore, as highlighted by Majewska et al. [21], RSCEs possess low cytotoxicity, high biocompatibility, and effective cellular uptake characteristics, making them promising agents for dermatological applications. Their plant origin not only addresses regulatory concerns but may also provide advantages in terms of scalability, consistency, and cost effectiveness compared to human-derived alternatives.

Exosomes derived from *Rosa damascena* stem cells represent a novel and biologically active therapeutic modality for wound healing and scar regeneration. *Rosa damascena* is well documented to possess a broad spectrum of pharmacological effects, including antioxidant, anti-inflammatory, antimicrobial, and regenerative properties, largely attributed to its rich composition of flavonoids (such as quercetin and kaempferol), phenolic compounds, anthocyanins, and vitamin C [22]. These bioactive constituents have been shown to modulate MAPK and GLK signaling pathways, enhance dermal hydration and tissue resilience [23,24], and inhibit oxidative stress [25]. Given that exosomes serve as natural carriers of proteins, lipids, and nucleic acids that reflect the therapeutic profile of their parent cells, exosomes derived from *Rosa damascena* stem cells are likely to recapitulate and amplify the plant’s intrinsic healing mechanisms at the cellular level. By promoting fibroblast activation, angiogenesis, and collagen remodeling, while reducing inflammatory cytokines and reactive oxygen species, these plant-derived exosomes offer a cell-free, non-immunogenic, and stable alternative to traditional growth factor therapies in the treatment of cutaneous wounds and hypertrophic scars.

### 3.1. Mechanisms Underlying Observed Clinical Improvements

The clinical improvements documented in our cases likely reflect the biological activities of plant-derived exosome-like nanoparticles that have been reported in the literature. Several mechanisms may contribute to the observed effects.

#### 3.1.1. Enhanced Cellular Proliferation and Migration

Similar to findings by Natania et al., who demonstrated that *Physalis peruviana*-derived exosome-like nanoparticles (PENCs) induced human dermal fibroblast proliferation and migration [8], the RSCEs used in our cases may have stimulated cellular regeneration in the treated areas. This proliferative effect is critical for wound healing and scar remodeling, potentially explaining the progressive improvement observed over successive treatments.

The same mechanisms have been reported with other plant-derived nanoparticles. For instance, ginseng-derived nanoparticles (GDNPs) were shown by Yang et al. [9] to enhance the migration in HaCaT cells and HUVECs and increase the secretion of MMP-1, fibronectin-1, elastin-1, and COL1A1, indicating potential mechanisms for the effect of PDENs on wound healing. Similarly, Şahin et al. [10] demonstrated that wheat-derived exosomes yielded remarkable proliferative and migratory effects on endothelial, epithelial, and dermal fibroblast cells.

#### 3.1.2. Modulation of Extracellular Matrix Remodeling

The Natania study also found that PENCs upregulated collagen I production while reducing MMP-1 levels [8], suggesting a role in extracellular matrix regulation. Similar mechanisms may have been at work in our cases, particularly in the dog bite scar where improved texture and flattening were observed. The improved appearance of scars in our cases aligns with the findings by Lei et al., who reported that specific exosome-like vesicles accelerated burn wound healing and ameliorated scarring [7].

This mechanism was substantiated by Li et al. [16], who investigated the effect of ADSC-Exos on the expression of ECM-related genes in skin fibroblasts. They found that ADSC-Exos can regulate the ratio of fibroblast Col-III to Col-I, TGF-3 to TGF-1, and MMP3 to TIMP-1, as well as regulate fibroblast differentiation to affect ECM reconstruction, thereby alleviating scar formation. Interaction with the extracellular matrix is critical for reducing scar visibility and achieving the kind of improvements we observed in our clinical cases.

#### 3.1.3. Anti-Inflammatory and Antioxidant Effects

Several studies have documented the anti-inflammatory and antioxidant properties of plant-derived exosome-like nanoparticles (PDENs). Kim et al. [26] reported on the antioxidant effects of small extracellular vesicles derived from *Aloe vera* peels in wound healing, while Perut et al. [27] found that strawberry-derived exosome-like nanoparticles prevented oxidative stress in human mesenchymal stromal cells. As reported by Wu et al. [11], PDENs promote the healing of chronic wounds through mechanisms such as enhancing cell proliferation and migration, exerting anti-inflammatory effects, promoting angiogenesis, modulating immune responses, and providing antibacterial activity. For instance, aloe vera-derived nanovesicles not only promoted the proliferation and migration of human keratinocytes and fibroblasts but also reduced reactive oxygen species (ROS) levels in keratinocytes, supporting their role in oxidative stress modulation and tissue repair [26]. The reduction in erythema observed in our cases, particularly in the burn and dog bite scars, may reflect these anti-inflammatory effects.

Recent work further supports the bioactivity of PDENs from other botanical sources. Zhu and He [28] demonstrated that ginger-derived exosome-like nanoparticles suppressed NF-κB-mediated inflammation and scavenged ROS, highlighting their potential in controlling oxidative stress-related tissue damage. Likewise, Zhao et al. [29] showed that blueberry-derived exosome-like nanoparticles alleviated mitochondrial oxidative stress and lipid peroxidation in hepatocytes, ameliorating nonalcoholic fatty liver disease. These findings align with earlier results from Ju et al. [30], who reported that grape-derived exosomes protected intestinal tissue and stimulated stem cell regeneration in colitis models. Together, this growing body of evidence reinforces the potential of PDENs as safe, biocompatible, and effective therapeutic agents with intrinsic anti-inflammatory and antioxidant properties. When derived from plants like *Rosa damascena*, which is already known for its high content of polyphenols, flavonoids, and vitamin C [22], these vesicles may offer enhanced efficacy in promoting dermal regeneration and treating chronic or complex skin wounds.

#### 3.1.4. Synergistic Effects with Physical Treatments

The combination of RSCEs with microneedling (MN) and laser therapy likely produced synergistic effects. Microneedling serves as a critical component in our treatment approach, functioning as both a therapeutic modality and an effective delivery system for RSCEs. The controlled micro-injuries created by microneedling stimulate the skin’s natural wound healing cascade, initiating the release of growth factors that promote collagen and elastin production.

As noted by Lev-Tov [31], “small needles make a big difference” in cutaneous drug delivery, with microneedling enabling the passage of higher molecular weight therapeutics that would otherwise be excluded from percutaneous absorption. This temporary disruption of the skin barrier allows RSCEs to reach deeper dermal layers where they can interact with fibroblasts and other target cells.

The mechanical stimulation from microneedling itself initiates transforming growth factor-β (TGF-β) signaling and other regenerative pathways that work synergistically with the bioactive components of the exosomes. This dual-action approach—mechanical stimulation plus biological signaling—appears to enhance the effectiveness of treatment beyond what either component could achieve independently.

Microneedling also provides precise control over the depth and area of treatment, allowing customization based on scar type and location. In our protocol, needle depths of 0.2–0.4 mm were carefully selected to optimize exosome delivery while minimizing patient discomfort and recovery time. This controlled approach is particularly important for facial applications, where patient comfort and minimal downtime are significant considerations.

The combination of microneedling (MN) and exosome-based therapy represents an innovative strategy to enhance cutaneous regeneration and transdermal delivery of therapeutic agents. As outlined by Lim and Kim [32], MN transiently disrupts the stratum corneum, creating uniform microchannels that facilitate the permeation of bioactive compounds into deeper layers of the skin or systemic circulation, while maintaining a favorable safety and tolerability profile. These micro-injuries further stimulate dermal remodeling by promoting collagen and elastin synthesis. Zhang et al. [33] similarly emphasized that the microneedling-induced microenvironment offers a favorable milieu for the delivery of high-molecular-weight or otherwise skin-impermeable agents. Exosomes—nano-sized extracellular vesicles rich in growth factors, cytokines, and miRNAs—can leverage these transient microchannels to reach dermal targets with improved efficiency and stability. Their intrinsic regenerative and anti-inflammatory properties complement the wound-healing cascade initiated by MN, potentially enhancing clinical outcomes in scar revision, and chronic dermatologic conditions. Integrating MN-assisted delivery with exosome therapy thus provides a synergistic platform that overcomes the limitations of passive topical application while supporting sustained, localized, and biologically active treatment delivery [13].

Similarly, laser treatment may facilitate exosome penetration while also providing direct thermal effects on collagen remodeling. Laser-assisted drug delivery (LADD) has emerged as a particularly effective method for enhancing topical drug penetration into the skin. Wenande et al. [34] note that ablative fractional lasers exert an ablative effect on exposed skin and can significantly alter the stratum corneum’s physicochemical restrictions on topical uptake, enabling delivery of even extremely hydrophilic and high molecular weight drugs. This occurs through the application of Fick’s law, where laser treatment influences the permeability coefficient by increasing intracutaneous drug diffusivity after partial removal of stratum corneum layers.

Evidence-based clinical practice guidelines for LADD developed by Labadie et al. [35] support the safety and effectiveness of this approach in various medical and cosmetic settings. According to these guidelines, LADD is an effective treatment for actinic keratosis, cutaneous squamous cell carcinoma in situ, actinic cheilitis, hypertrophic scars, and keloids. Liedtke et al. [36] further confirmed that the combined use of exosome gel with CO_2_ laser resurfacing provided synergistic effects on both the efficacy and safety of atrophic acne scarring treatments.

Kwon et al. [14] demonstrated that the combined use of human adipose tissue stem cell-derived exosomes with fractional CO_2_ laser for treating acne scars yielded more favorable responses, a shorter recovery time, and fewer side effects. Their double-blind, randomized, split-face study showed that ASCE-treated sides achieved significantly greater improvements than control sides, suggesting that this multimodal approach may explain the significant improvements observed over relatively short treatment periods in our study as well.

The synergistic mechanisms behind the combination of fractional thulium laser with exosomes were further supported by Li et al. [37], who showed that the 1927 nm fractional thulium fiber laser (FTL) significantly improved epidermal thickness, melanin index, skin elasticity, and wrinkles in patients with photoaging. They proposed that FTL has a moderate affinity for water-containing tissues, which prevents epidermal turnover rather than causing it, while still being able to penetrate deep into 200–300 μm tissue depths and stimulate modest collagen regeneration. This unique property may be particularly beneficial when combined with exosome therapy for scar treatment.

#### 3.1.5. Standalone Topical Application

An important finding from our case series is the efficacy of RSCEs when applied as a standalone topical treatment, as demonstrated in the facial laser complication case. This patient achieved a 75% improvement in mVSS score and approximately 70% improvement in POSAS scores using only topical exosome application, without any device-assisted delivery methods. This observation suggests that while combination therapy with microneedling or laser may enhance outcomes in many cases, plant-derived exosomes possess intrinsic therapeutic properties that can be effective even without physical enhancement of penetration. This finding has significant clinical implications, particularly for patients who may not be candidates for device-based treatments due to medical contraindications, previous adverse reactions, or patient preference.

It is noteworthy that these mechanisms—enhanced cellular proliferation, modulation of extracellular matrix, and anti-inflammatory effects—appear to be active even with simple topical application, as evidenced by the fourth case in our series. This suggests that RSCEs may possess sufficient bioavailability and penetration capacity to exert therapeutic effects without requiring physical enhancement methods, although combination approaches may amplify these benefits.

### 3.2. Advantages of Plant-Derived Exosomes in Clinical Application

The use of plant-derived exosome-like nanoparticles offers several potential advantages over other regenerative approaches. As noted by Subha et al. [3], plant-derived exosome-like nanovesicles are easily sourced and relatively cost effective to produce, making them potentially more accessible for clinical use compared to mammalian-derived exosomes. Plant-derived exosomes have been reported to exhibit low immunogenicity [3], which may contribute to the absence of adverse reactions observed in our cases. Our cases demonstrated that RSCEs could be effectively delivered through various methods (topical application with microneedling and/or laser treatments), suggesting flexible integration into existing treatment protocols. The combination of exosomes with microneedling and laser therapy appeared to yield enhanced results, indicating that plant-derived exosomes can be effectively incorporated into multifaceted treatment approaches.

Plant-derived exosome-like nanoparticles (PDENs) exhibit several distinct advantages over mammalian-derived exosomes, particularly in the context of dermatological and cosmetic applications. PDENs offer high biosafety, minimal immunogenicity, and a favorable regulatory profile due to their natural, edible plant origins [38,39,40,41]. They can be produced at scale using cost-effective, standardized plant cell cultures under GMP-compliant conditions without the need for animal-derived components. Additionally, their robust lipid bilayer structure allows for improved physical stability and longer shelf life, making them especially well suited for topical formulations and commercial distribution in aesthetic medicine and skincare.

### 3.3. Limitations and Future Directions

The present case series provides valuable clinical insights, although several considerations should be acknowledged:

1. Preliminary Nature of Findings: As an initial clinical observation series, our study demonstrates promising outcomes across different scar types. While these results are encouraging, larger cohort studies would further substantiate these findings.
2. The Absence of a Control Group limits the ability to isolate the contribution of RSCEs; therefore, the results should be interpreted with caution, and controlled studies are necessary to confirm efficacy.
3. Age Range of Participants: The age range in our case series was limited to 34 to 52 years; future studies should include a broader spectrum of patient ages to determine whether the treatment is equally effective and safe across diverse age groups.
4. Combined Treatment Approach: Our protocol utilized RSCEs in conjunction with established physical modalities (microneedling and laser therapy). This multimodal approach reflects practical clinical care but makes it challenging to isolate the specific contribution of each component. The apparent synergy between these treatments warrants further exploration.
5. Composition of Preparations: The RSCE solution and ExoBalm used in our protocol contain additional bioactive ingredients alongside exosomes. While exosomes are believed to play a central regenerative role, the presence of these complementary components makes it challenging to isolate the specific contribution of exosomes alone to the observed outcomes.
6. Personalized Treatment Parameters: Treatment protocols were customized based on each patient’s unique scar characteristics and clinical response. While this personalization represents sound clinical practice, standardized protocols would facilitate more direct comparisons in future research.
7. Clinical Focus: As practicing clinicians, our primary focus was on documenting treatment outcomes using validated clinical assessment tools. More detailed characterization of the molecular and cellular mechanisms of RSCEs, while valuable, was beyond the scope of this clinical observation.

Future directions could include:

- Expanded patient cohorts with diverse scar etiologies to confirm the consistency of outcomes;
- Comparative studies evaluating RSCEs with and without complementary physical treatments;
- Refinement of treatment protocols for specific scar types.

This is the first clinical case series demonstrating objective, standardized scar improvement outcomes using plant-derived exosome-like nanoparticles combined with established scar treatments. Unlike previous animal or in vitro studies, our work provides initial human data supporting RSCE application in diverse scar etiologies.

The promising results observed in this case series suggest that plant-derived exosome-like nanoparticles represent a valuable addition to the clinical armamentarium for scar management. The combination of objective improvement in validated scar scales, excellent safety profile, and positive patient satisfaction warrants continued clinical investigation of this approach.

As indicated by Li et al. [16], the effects of adipose-derived stem cells and their exosomes in wound healing and scar prevention involve complex mechanisms that remain incompletely understood. Precision therapy may require understanding how these exosomes affect fibroblasts and myofibroblasts at different stages of scar formation. The role of exosomes in the different stages of scar formation deserves further study, as does the effect of these therapies on fibroblasts from different host tissues, which can show considerable functional differences, as noted by Li et al. [18].

## 4. Materials and Methods

### 4.1. Study Design and Ethics

This retrospective case series examines the outcomes of wound healing and scar treatment using plant-derived exosome-like nanoparticles (specifically RSCEs) in combination with microneedling and laser therapy.

All treatments were performed in accordance with the Declaration of Helsinki, and written informed consent form was obtained from all patients for both treatment and the use of their clinical images and case details for research and publication purposes. The study received ethical approval from the Bioethics Committee at the District Medical Chamber in Krakow (L.dz. OIL/KBL/12/2025).

### 4.2. Rose Stem Cell Exosomes—Exosomal Solution and Exosomal Balm

The *Rosa damascena* stem cell-derived exosome preparation used in this study (ExoSCRT™, ExoCoBio Inc., Seoul, Republic of Korea) was manufactured from standardized plant callus cultures and purified using tangential flow filtration (TFF). According to the manufacturer’s technical documentation and quality control reports, the final product underwent validated sterility testing, particle size characterization by nanoparticle tracking analysis (mode: 95–115 nm), and detection of exosomal surface markers (CD9, CD63, and CD81) by western blot. As these markers are typically associated with mammalian exosomes and were not independently validated in this study, we relied on the quality control data provided by the manufacturer [42,43]. These specifications ensure batch-to-batch consistency and support the biological integrity of the exosome preparation.

The RSCE solution used in this study was ASCEplus Derma Signal Kit SRLV (ExoCoBio Inc., Seoul, Republic of Korea), a sterile, ready-to-use formulation containing approximately 5 billion *Rosa damascena* callus-derived exosomes (RSCEs) per vial. According to the manufacturer, RSCEs constitute ~0.16% of the total formulation by weight. The solution also includes other components, such as hyaluronic acid, amino acids, peptides, and vitamins, commonly found in skincare formulations. Based on preclinical data, RSCEs are considered the primary active ingredient responsible for regenerative effects. The solution was stored at 2–8 °C and prepared ex tempore immediately prior to topical application. RSCEs were applied during microneedling and/or laser procedures, or as monotherapy.

In addition to the exosomal solution, ExoBalm (ExoCoBio Inc., Seoul, Republic of Korea) was applied post-procedure. ExoBalm is a cream formulation containing approximately 2.5 billion RSCEs per application combined with complementary active ingredients, including tranexamic acid (2%), madecassoside (0.5%), panthenol (1%), and niacinamide (2%), to support skin recovery. All RSCE formulations used in this study are commercially available cosmetic products with manufacturer-documented sterility, bioactivity, and preclinical safety profiles in in vitro and in animal models. We did not conduct additional preclinical tests or biomolecular analyses. No adverse effects were observed in any patient.

#### 4.2.1. Treatment Protocols—Dog Bite

A 34-year-old female patient with a scar resulting from a dog bite underwent a series of four treatments spanning from January 2025 to April 2025. The scar was located in the philtrum area, measured approximately 2.5 cm in length, and presented with noticeable erythema, elevation, and textural irregularity. During the first treatment session on 31 January 2025, the patient received microneedling using a Dermapen 4.0 at needle depths of 0.2–0.4 mm, with topical application of RSCEs (0.5 mL) during the procedure. The second treatment, performed on 28 February 2025, combined thulium laser treatment (Lutronic Ultra MD) with specific settings for scar tissue (10 J, 4 passes), followed by microneedling using a Dermapen 4.0 at needle depths of 0.2–0.4 mm and topical application of RSCEs (0.5 mL). The third treatment on 25 March 2025 repeated the protocol from the second session with identical laser settings, microneedling parameters, and exosome application. The fourth and final treatment, conducted on 18 April 2025, comprised thulium laser treatment (10 J, 4 passes) and scar acupuncture with topical application of RSCEs (0.5 mL). After each treatment session, the remaining RSCE solution (approximately 4.5 mL) was dispensed to the patient for home use, with instructions to store it in the fridge and apply it to the treatment area 4–5 times daily, which provided sufficient coverage for approximately 2 days. Additionally, starting from the second day post-treatment, ExoBalm was introduced into the home care regimen, with instructions for twice-daily application (morning and evening) to enhance the healing process and optimize treatment outcomes. The interval between treatments was approximately 4 weeks to allow for proper healing and integration of the therapeutic effects. Treatment outcomes were documented through clinical photography and assessed using the modified Vancouver Scar Scale (mVSS) and the Patient and Observer Scar Assessment Scale (POSAS).

#### 4.2.2. Treatment Protocols—Forehead Trauma

A patient with a forehead scar resulting from a car accident underwent a series of three treatments. The scar presented as raised, erythematous, and displayed irregular texture. During the first treatment session on 30 December 2024, the patient received thulium laser therapy using Lutronic Ultra (12 J, 4 passes) combined with microneedling using a Dermapen 4.0 at needle depths of 0.2–0.4 mm, with topical application of RSCEs. Additionally, scar acupuncture was performed at a depth of 0.2 mm using a SOMA 0.30 × 30 mm needle. The second treatment, performed on 28 January 2025, utilized thulium laser treatment with increased energy (Lutronic Ultra, 13 J, 4 passes), followed by the same microneedling protocol with RSCEs and scar acupuncture. The third treatment on 5 March 2025 further increased the laser energy to 14 J while maintaining the same protocol with a Dermapen 4.0 and RSCEs. After each treatment session, the remaining RSCE solution (approximately 4.5 mL) was dispensed to the patient for home use, with instructions to store it in the fridge and apply it to the treatment area 4–5 times daily, which provided sufficient coverage for approximately 2 days. Additionally, starting from the second day post-treatment, ExoBalm was introduced into the home care regimen, with instructions for twice-daily application (morning and evening) to enhance the healing process and optimize treatment outcomes. Treatment outcomes were documented through clinical photography and assessed using the mVSS and POSAS to provide comprehensive objective and subjective evaluation of scar improvement.

#### 4.2.3. Treatment Protocols—Hot Oil Burn

The patient had a burn injury caused by hot cooking oil spilled directly on the thigh. The initial injury presented with significant erythema, blistering, and damaged skin integrity. The patient began a series of four treatments starting on 31 October 2024. The first treatment involved thulium laser therapy (Lutronic Ultra MD, 8 J, 4 passes) combined with microneedling using a Dermapen 4.0 at needle depths of 0.2–0.4 mm and topical application of RSCEs (1 mL). The second treatment on 28 November 2024 increased the laser energy to 10 J while maintaining the same microneedling parameters and exosome application. For the third session on 20 December 2024, the laser energy was further increased to 12 J with continued microneedling and exosome application. The final treatment on 10 February 2025 maintained the same parameters as the third session. Post-treatment care included moisturization, sun protection, and avoiding friction to the treated area. After each treatment session, the remaining RSCE solution (approximately 4 mL) was dispensed to the patient for home use, with instructions to store it in the fridge and apply it to the treatment area 4 times daily, which provided sufficient coverage for approximately 1 day. Additionally, starting from the second day post-treatment, ExoBalm was introduced into the home care regimen, with instructions for twice-daily application (morning and evening) to enhance the healing process and optimize treatment outcomes. Treatment outcomes were documented through clinical photography taken at baseline and after each treatment session. Assessment employed mVSS and POSAS for objective evaluation of treatment efficacy, enabling quantitative comparison with the other scar types treated in this series.

#### 4.2.4. Treatment Protocols—Facial Laser Treatment Complications

A 52-year-old female patient presented with significant iatrogenic complications following a combined erbium-YAG and thulium laser treatment performed for skin quality improvement. The patient experienced immediate adverse effects, including severe erythema, edema, and textural irregularities across the right cheek area. Within 24 h post-procedure, burn blisters developed, followed by wound formation along the jawline within several days, indicating substantial tissue damage beyond typical post-procedural reactions. The patient underwent a multi-stage treatment approach. Initial management from 1 February to 19 March 2025 consisted of conventional wound care using topical antibiotics (Fusacid H), corticosteroids (Pimafucort) for one day, then hydrogel dressings together with manuka honey for the next 7 days, and subsequently post-inflammatory hyperpigmentation treatments in the form of commercially available creams (Alantan Plus, La Roche Posay Mela B3). On day 8 post-injury, injectable I-PRF (Injectable Platelet-Rich Fibrin) was administered to promote tissue regeneration. This conventional approach yielded limited improvement.

At six weeks post-injury (19 March 2025), she was referred to our practice. RSCEs were applied as a topical mask for three consecutive days. Notably, the patient declined device-based interventions (microneedling or laser therapy) due to concerns about further complications and poor psychological condition. The patient was provided with ExoBalm for twice-daily home application. At the follow-up appointment on 10 April 2025, clinical improvement was deemed sufficient to forego the planned Dermapen treatment.

The final assessment on 9 May 2025 showed marked improvement, with the mVSS score reduced from 8/13 to 2/13 (75% improvement) and the POSAS scores improving from 42/60 (Observer) and 47/60 (Patient) to 13/60 and 16/60, respectively (~70% improvement). The treatment outcome was documented through photography and objective assessment scales, demonstrating the efficacy of RSCEs as a standalone topical treatment without device assistance.

### 4.3. Scar Assessment and Literature Review Methodology

Treatment outcomes were documented through clinical photography taken before each treatment session and at the final follow-up. For all cases, we employed the modified Vancouver Scar Scale (mVSS) to objectively assess the progression of scar improvement. The mVSS evaluates four parameters: vascularity (0 = normal, 1 = pink, 2 = red, 3 = purple), pigmentation (0 = normal, 1 = hypopigmentation, 2 = hyperpigmentation), pliability (0 = normal, 1 = supple, 2 = yielding, 3 = firm, 4 = ropes, 5 = contracture), and height (0 = flat, 1 = <2 mm, 2 = 2–5 mm, 3 = >5 mm). The total mVSS score ranges from 0 (normal skin) to 13 (worst scar condition). Assessments were performed by two independent clinicians before each treatment session, and the average scores were recorded.

Scar assessments were performed independently by two experienced clinicians trained in using the POSAS and mVSS scoring systems. Prior to evaluation, both assessors participated in calibration sessions using standardized reference images to harmonize scoring interpretation. In cases of minor disagreement, final scores were established by consensus.

Additionally, the Patient and Observer Scar Assessment Scale (POSAS) was used to incorporate the patient’s subjective experience. This scale includes both observer and patient components: the observer scale evaluates vascularization, pigmentation, thickness, relief, pliability, and surface area, while the patient scale assesses pain, itching, color, stiffness, thickness, and irregularity. Each parameter is scored from 1 (normal skin) to 10 (worst imaginable scar), with the total score ranging from 6 to 60 for each component and lower scores indicating better outcomes. For the burn and forehead trauma cases, evaluation focused on scar appearance (color, texture, and relief), scar size and thickness, skin flexibility in the treated area, and patient-reported satisfaction with the cosmetic result.

A comprehensive literature search was conducted to identify relevant studies on plant-derived exosome-like nanoparticles in wound healing and scar treatment. The search included, but was not limited to, the following databases: PubMed, Scopus, and Web of Science. Key search terms included “plant exosomes,” “exosome-like nanoparticles,” “plant-derived nanovesicles,” “wound healing,” “skin regeneration,” and “scar treatment.” Studies were included if they presented original research on plant-derived exosome-like nanoparticles with potential applications in wound healing, skin regeneration, or related fields. The review particularly focused on studies published between 2018 and 2025 to ensure relevance to current scientific understanding and clinical practice.

While the review was not conducted according to systematic review guidelines such as PRISMA, efforts were made to ensure comprehensive coverage of the most relevant and recent studies. Titles and abstracts were screened manually for relevance, and full-text articles were assessed for scientific rigor and applicability to the clinical aims of this study.

Patients were included if they presented with visible scars persisting at least several weeks post-injury or procedure (minimum 1 week), had no active infections or uncontrolled systemic diseases, and provided informed consent. No patients were excluded after recruitment. Outcomes varied slightly depending on scar etiology and patient adherence to home care instructions.

## 5. Conclusions

This case series provides preliminary evidence supporting the potential efficacy of plant-derived exosome-like nanoparticles, specifically RSCEs, in the treatment of various types of scars when combined with microneedling and laser therapy, or as a standalone topical treatment. The documented objective improvements, measured using standardized scar assessment scales, along with clinical enhancements in scar appearance, texture, and coloration across different scar etiologies—dog bite, burn, traumatic injury, and iatrogenic laser damage—suggest that this approach may offer a valuable addition to the current range of scar management strategies.

The observed clinical improvements align with findings in the existing literature on plant-derived exosomes, which have demonstrated capabilities to enhance cellular proliferation, modulate extracellular matrix remodeling, and exert anti-inflammatory effects. The apparent synergy between exosome therapy and established physical treatments (microneedling and laser) highlights the potential benefits of multimodal approaches to scar management. Notably, the successful treatment of iatrogenic laser-induced complications using only topical exosome application demonstrates that significant clinical improvements can be achieved even without device-based interventions.

Plant-derived exosome-like nanoparticles represent a promising frontier in wound healing and scar treatment. Their relative accessibility, cost effectiveness, and favorable safety profile make them particularly attractive for clinical translation. As our understanding of their biological activities continues to evolve, plant-derived exosomes may become an increasingly important component of comprehensive approaches to wound management and scar mitigation.

While limitations in study design preclude definitive conclusions about the specific contribution of exosomes to the observed outcomes, the consistent pattern of improvement across cases warrants further investigation through more rigorous experimental approaches. Future research should focus on elucidating the precise mechanisms of action, optimizing treatment protocols, and comparing the efficacy of different plant-derived exosome preparations.

## Figures and Tables

**Table 1:** Modified Vancouver Scar Scale (mVSS) assessment throughout treatment course.

Parameter	Baseline	4 Weeks	8 Weeks	12 Weeks	Description
Vascularity	2	2	1	1	0 = normal, 1 = pink, 2 = red, 3 = purple
Pigmentation	0	0	0	0	0 = normal, 1 = hypopigmentation, 2 = hyperpigmentation
Pliability	3	2	1	1	0 = normal, 1 = supple, 2 = yielding, 3 = firm, 4 = ropes, 5 = contracture
Height	2	1	1	0	0 = flat, 1 = <2 mm, 2 = 2–5 mm, 3 = >5 mm
Total mVSS Score	7/13	5/13	3/13	2/13	Lower score indicates better outcome
Improvement (%)	–	29%	57%	71%	Percentage improvement from baseline

**Table 2:** Patient and Observer Scar Assessment Scale (POSAS) throughout treatment course.

POSAS Component	Baseline	4 Weeks	8 Weeks	12 Weeks	Score Range
Observer Score	32/60	26/60	18/60	14/60	6–60 (lower is better)
Patient Score	41/60	35/60	22/60	16/60	6–60 (lower is better)
Observer Improvement (%)	–	19%	44%	56%	Percentage improvement from baseline
Patient Improvement (%)	–	15%	46%	61%	Percentage improvement from baseline

**Table 3:** Modified Vancouver Scar Scale (mVSS) assessment for forehead trauma throughout treatment Course.

Parameter	Baseline (30 December 2024)	After 1st Treatment (28 January 2025)	After 2nd Treatment (5 March 2025)	After 3rd Treatment (Final)
Vascularity	2 (red)	2 (red)	1 (pink)	1 (pink)
Pigmentation	0 (normal)	0 (normal)	0 (normal)	0 (normal)
Pliability	3 (firm)	2 (yielding)	2 (yielding)	1 (supple)
Height	2 (2–5 mm)	1 (<2 mm)	1 (<2 mm)	0 (flat)
Total mVSS score	7/13	5/13	4/13	2/13
Improvement from baseline	–	29%	43%	71%

**Table 4:** Patient and Observer Scar Assessment Scale (POSAS) throughout treatment course.

POSAS Component	Baseline (30 December 2024)	After 1st Treatment (28 January 2025)	After 2nd Treatment (5 March 2025)	After 3rd Treatment (Final)
Observer Score (out of 60)	33	28	24	15
Patient Score (out of 60)	42	36	31	18
Observer improvement from baseline	–	15%	27%	55%
Patient improvement from baseline	–	14%	26%	57%

**Table 5:** Modified Vancouver Scar Scale (mVSS) assessment for hot oil burn.

Parameter	Baseline (26 October 2024)	After 1st Treatment (31 October 2024)	After 2nd Treatment (28 November 2024)	After 3rd Treatment (20 December 2024)	After 4th Treatment (10 February 2025)
Vascularity	3 (purple)	2 (red)	2 (red)	1 (pink)	1 (pink)
Pigmentation	1 (hypopigmentation)	1 (hypopigmentation)	1 (hypopigmentation)	0 (normal)	0 (normal)
Pliability	3 (firm)	3 (firm)	2 (yielding)	1 (supple)	1 (supple)
Height	2 (2–5 mm)	2 (2–5 mm)	1 (<2 mm)	1 (<2 mm)	0 (flat)
Total mVSS score	9/13	8/13	6/13	3/13	2/13
Improvement from baseline	–	11%	33%	67%	78%

**Table 6:** Patient and Observer Scar Assessment Scale (POSAS) for hot oil burn.

POSAS Component	Baseline (26 October 2024)	After 1st Treatment (31 October 2024)	After 2nd Treatment (28 November 2024)	After 3rd Treatment (20 December 2024)	After 4th Treatment (10 February 2025)
Observer Score (out of 60)	45	40	32	22	11
Patient Score (out of 60)	49	43	37	25	14
Observer improvement from baseline	–	11%	29%	51%	76%
Patient improvement from baseline	–	12%	24%	49%	71%

**Table 7:** Modified Vancouver Scar Scale (mVSS) assessment for facial laser treatment complications.

Assessment Date	Vascularity (0–3)	Pigmentation (0–2)	Pliability (0–5)	Height (0–3)	Total Score (0–13)
Initial (1 February 2025)	3 (purple)	1 (hypopigmentation)	3 (firm)	1 (<2mm)	8
2 weeks (15 February 2025)	2 (red)	1 (hypopigmentation)	3 (firm)	1 (<2mm)	7
4 weeks (1 March 2025)	2 (red)	2 (hyperpigmentation)	2 (yielding)	1 (<2mm)	7
6 weeks (19 March 2025)	2 (red)	2 (hyperpigmentation)	2 (yielding)	0 (flat)	6
8 weeks (10 April 2025)	1 (pink)	1 (hypopigmentation)	1 (supple)	0 (flat)	3
Final (1 May 2025)	1 (pink)	0 (normal)	1 (supple)	0 (flat)	2

**Table 8:** Patient and Observer Scar Assessment Scale (POSAS) for facial laser treatment complications.

Assessment Date	Observer Score (6–60)	Patient Score (6–60)	Combined Score (12–120)	% Improvement from Baseline
Initial (1 February 2025)	42	47	89	–
2 weeks (15 February 2025)	38	43	81	9%
4 weeks (1 March 2025)	36	40	76	15%
6 weeks (19 March 2025)	30	35	65	27%
8 weeks (10 April 2025)	19	23	42	53%
Final (1 May 2025)	13	16	29	67%

## Data Availability

Data is contained in the paper.
